# Evaluation of recurrent *GNPTAB*, *GNPTG*, and *NAGPA* variants associated with stuttering

**DOI:** 10.1002/ggn2.10043

**Published:** 2021-05-20

**Authors:** Nandhini Devi Gunasekaran, Chandru Jayasankaran, Jeffrey Justin Margret, Mathuravalli Krishnamoorthy, C. R. Srikumari Srisailapathy

**Affiliations:** ^1^ Department of Genetics, Post Graduate Institute of Basic Medical Sciences University of Madras, Taramani Campus Chennai India; ^2^ LifeBytes India Pvt. Ltd Bengaluru Karnataka India

**Keywords:** candidate genes, *GNPTAB*, *GNPTG*, language fluency, *NAGPA*, neurodevelopmental genetics, stuttering

## Abstract

Stuttering is a childhood‐onset fluency disorder, intertwined with physiological, emotional, and anxiety factors. The present study was designed to evaluate the recurrence of the reported mutations among three previously implicated (*GNPTAB*, *GNPTG*, *NAGPA*) candidate genes, in persons with stuttering from south India. Mutation screening was performed among 64 probands on 12 specific exons, by Sanger sequencing. A total of 12 variants were identified, which included five nonsynonymous, five synonymous, and two noncoding variants. Three unrelated probands harbored heterozygous missense variants at conserved coding positions across species (p. Glu1200Lys in *GNPTAB*, p. Ile268Leu in *GNPTG* and p. Arg44Pro in *NAGPA*). Of these, only one variant (p. Glu1200Lys in *GNPTAB*) cosegregated with the affected status while p. Ile268Leu in *GNPTG* gene was found to be a rare de novo variant. Although this study identified some previously reported variants that have been claimed to have a role in stuttering, we confirmed only one of these to be a likely causal de novo variant (p.Ile268Leu) in the *GNPTG* gene at an allele frequency of 0.8% (1/128) in the families with stuttering.

## Graphical abstract

Screening 64 individuals with stuttering from South India, we evaluated the recurrence of a total of 12 variants in three previously implicated (*GNPTAB*, *GNPTG*, *NAGPA*) candidate genes. This upset plot shows the combinations of synonymous and nonsynonymous variants among the probands in three genes implicated in stuttering.

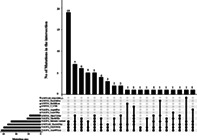

## 1 INTRODUCTION

Speech is a unique motor function and when affected causes both receptive and expressive communication disorders, significantly reducing the quality of life.^28^ Stuttering is an expressive fluency disorder, characterized by repetitions, prolongations, blocks, along with secondary behaviors (head jerks, lip tremors, and eye blinks) and often lead to psychological problems such as increasing anxiety.^1^

Developmental stuttering arises in children of the 2 to 5 years age group, but most of them (80%) recover spontaneously. Males are more affected than females with a male to female ratio of ~5:1 and a greater chance of recovery was reported among females.^2^, ^3^, ^4^ The prevalence of stuttering ranges from 0.3% to 5.6%. The average prevalence over the lifespan may be lower than the commonly cited 1%.^5^ In a recent study based on 75 000 school children in south India, we reported a prevalence of 0.46%.^6^

Stuttering is a complex disorder caused by an interplay of genetic and environmental factors in a majority of cases, as evident from twin studies with variable heritability.^7^ Although familial aggregation was observed genetic epidemiological studies provided inconsistent evidence for the mode of inheritance, with varied reports on dominant, recessive, and sex‐modified inheritance.^8^, ^9^ Genetic dissection is challenging due to gene‐gene or gene‐environment interactions, genetic heterogeneity, gender bias, incomplete penetrance, and phenocopies.^10^ Initial linkage studies found suggestive evidence for 12 chromosome regions (1, 2q, 3q, 5q, 7q, 9p, 9q, 13q, 15q, 18p, 18q, 20p) that were implicated in stuttering but with little overlap across studies.^11^, ^12^, ^13^ However definitive evidence for linkage was identified on chromosomes 3, 12, and 16q in highly consanguineous Pakistani families^14^, ^15^, ^16^ and on chromosomes 2, 3, 14, and 15 in a single large Cameroon family.^17^ The analysis of these linked regions resulted in the identification of *GNPTAB* locus on chromosome 12^18^ and *AP4E1* locus on chromosome 15.^19^ Two other genes *GNPTG* and *NAGPA*, located on chromosome 16p, acting in the same pathway were also implicated as candidate genes for stuttering. These findings provide new opportunities to understand the biological basis of stuttering where all the genes identified point to intracellular trafficking deficits.^18^

*GNPTAB* and *GNPTG* genes together code for a phosphotransferase enzyme while *NAGPA* encodes an enzyme responsible for the removal of N‐acetylglucosamine thereby uncovering the mannose 6 phosphate (M6P) marker targeting acid hydrolases to lysosomes. Most of the variants in these genes were identified to be of missense type in persons with stuttering (PWS). However, family‐based linkage studies to date did not report any significant signal on chromosome 16p loci that bear *GNPTG* and *NAGPA* genes. Homozygous loss of function variants in *GNPTG* causes gamma‐type mucolipidosis III (MIM 252605) and *GNPTAB* causes alpha‐beta type mucolipidosis II (MIM 252500) and III (MIM 252600). These recessive rare fatal lysosomal storage disorders result from failure in the targeting of lysosomal enzymes to lysosomes. However, it was hypothesized that *GNPTAB* and *GNPTG* variations involved in stuttering differ from the—mostly truncating—mutations reported in mucolipidosis II and III.^7^

Although linkage studies are spread across Hutterite, European and American populations, variants identified by linkage in the four genes, *GNPTAB*, *GNPTG*, *NAGPA*, and *AP4E1*, are restricted to two regions (Pakistan^18^; Cameroon^19^) with distinct continental ancestry and language groups. However, follow‐up studies by the same group have studied the role of these genes in stuttering in European and American populations. Variants in these genes were estimated to be present in 20% of the unrelated stuttering individuals; 8% of these variants are also reported in public databases, likely carried by individuals who might not have been phenotyped for stuttering, who may be current or former stutterers.^7^

Since none of the genes identified have an obvious connection to speech, mouse model studies have linked the implicated genes to brain activity. First, the *GNPTAB* gene mutation (p. Glu1200Lys) was engineered in mice to observe any change in mice vocalization. Recording the ultrasonic mouse vocalization of pups showed intriguing patterns of gaps and pauses that are similar to human stuttering.^20^ The introduced mutation also results in the loss of astrocytes in the mouse corpus callosum that decelerates the communication between hemispheres by a small‐time lag that can be noticed only in fine motor performance.^21^ Indeed, genomics of stuttering is an emerging area of research interest where new genes are yet to be identified.

A GWAS study of stuttering investigated 84 subjects of age group ranging from 13 to 70 years of northern European ancestry, to identify candidate genes that influence the risk of developing stuttering. With limited statistical power, the study suggested 10 loci with a *P*‐value between 10^−4^ and 10^−8^, harboring candidate genes (*FADS2*, *PLXNA4*, *CTNNA3*, *ARNT2*, *EYA2*, *PCSK5*, *SLC24A3*, *FMN1*, *ADARB2*, and a noncoding RNA *RNU6‐259P*) involved in neural pathways.^22^

From the genetic perspective, the so far identified candidate genes are known to play a role in targeting enzymes to lysosomes that are crucial for biogenesis and in the maintenance of myelin sheaths. From neurological perspective hyperactivity of dopamine and the white matter abnormalities observed in stuttering, provide a possible neurochemical basis with a yet unclear mechanism. Owing to significant plasticity of the brain, the study was unable to account for the observed differences among PWS and control, as to whether they are cause or result of stuttering.^23^ Thus, the work of identifying the connections among dopamine, neural circuits, and protein turnover has just begun.

Until now, no studies from India implicate any genes for stuttering or have established the frequency of previously implicated variants. This gap motivated us first to establish the frequency of variants in the previously implicated genes for stuttering in our population, before initiating advanced approaches. Here, we evaluate the recurrence of the reported mutations among the three implicated (*GNPTAB*, *GNPTG*, *NAGPA)* stuttering candidate genes in PWS from South India. Attempts to employ identical experimental designs to concurrently verify and replicate the findings independently, on the one hand, help us understand population‐specific variations and, on the other hand, will facilitate the pooling of data for meta‐analysis.

## 2 RESULTS

### 2.1 Mutation analysis of the three putative genes in stuttering

A total of 64 unrelated probands with nonsyndromic persistent stuttering (sex ratio of 59 males: 5 females [12:1]; mean age at onset of 5.13 years) were screened for the recurrence of mutations in the three stuttering implicated genes. Sixty‐seven percent (43/64) of them had a family history. More than 50% of PWS were found to be severe; 53.1% severe (34/64), 28.1% moderate (18/64), and 18.8% mild (12/64).

**FIGURE 1:**
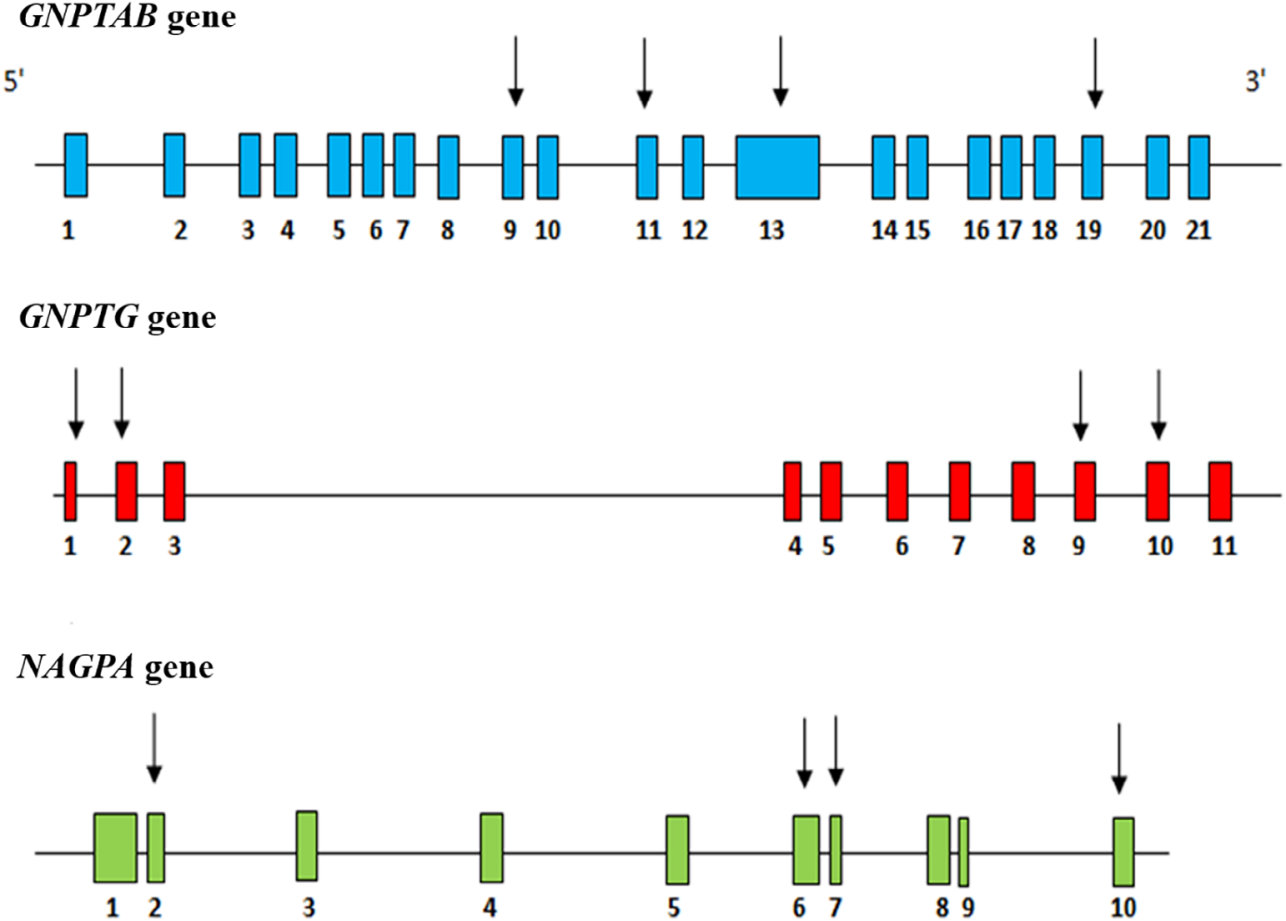
The 12 specific exons screened across the three genes implicated in stuttering

**FIGURE 2:**
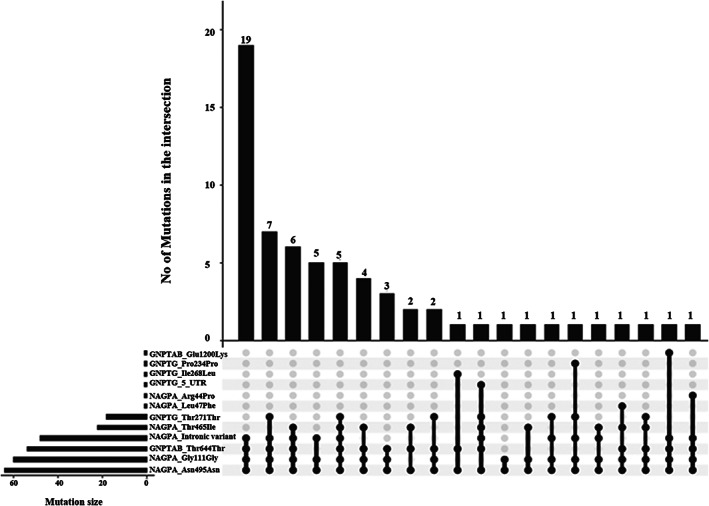
An upset plot of identified variants in this study across three genes implicated in stuttering to show combination of variants among probands. For each variant that is a part of intersection, a black filled circle is shown in the matrix and for the variant that is not part of intersection, a light gray circle is placed. The number of probands bearing the set of variants (column‐based relationships) is emphasized by vertical black line connecting the top most and bottom most black circle. The bar chart on top and left of the matrix gives the size of intersections and sets, respectively

Mutation screening of the 12 specific exons previously reported (Figure 1), identified a total of 12 variants that include 5 missense, 5 synonymous, and 2 noncoding variants (Tables 1 and 2; Supplementary Figures A1‐A3). The identified variants and their combinations were analyzed in an upset plot using the R program^24^ (Figure 2). The plot visualizes intersections of sets as a matrix in which the rows represent variants and the columns represent the number of probands having that particular combination of variants. Thus, each identified variant represents a set and each proband represents an element that is contained in one or more sets. We observed that all the probands had p. Asn495Asn synonymous variant in the *NAGPA* gene in common, along with at least two or more other synonymous variants. Variants like p. Glu1200Lys in *GNPTAB*, 5′ UTR (c.‐4 C > T), p. Pro234Pro and p. Ile268Leu in *GNPTG* and p. Arg44Pro and p. Leu47Phe in *NAGPA* gene, occurred singly in unrelated probands.

**TABLE 1:** Allele frequencies of the 12 variants observed in *GNPTAB*, *GNPTG*, and *NAGPA* genes among the 64 probands with stuttering and their comparison with gnomAD database

S. No.	GENE	Nucleotide change	Amino acid change	Exon	dbSNP ID	Hom/Het (n = 64 probands)	Allele frequency (n = 128 alleles)	Stuttering studies	Allele frequency in South Asian gnomAD	Allele frequency in total gnomAD
Missense variants										
1	*GNPTAB*	c.3598G > A	p.Glu1200Lys	Exon 19	rs137853825	‐/1	0.008	(8/1013) = 0.00789	0.0214	0.0028
2	*GNPTG*	c.802A > C	p.Ile268Leu	Exon 10	rs759796840	‐/1	0.008		0.00003266	0.000004
3	*NAGPA*	c.131G > C	p.Arg44pro	Exon 2	rs374266430	‐/1	0.008	(1/1013) = 0.000099	0.002851	0.0004
4	*NAGPA*	c.139C > T	p.Leu47Phe	Exon 2	rs371054576	‐/1	0.008		0.003873	0.0005
5	*NAGPA*	c.1394 C > T	p.Thr465Ile	Exon 10	rs7188856	‐/22	0.172		0.1751	0.2990
Synonymous variants										
6	*GNPTAB*	c.1932A > G	p.Thr644Thr	Exon13	rs10778148	42/12	0.75	120/1708 alleles	0.6236	0.5900
7	*GNPTG*	c.702 T > C	p.Pro234Pro	Exon 9	rs532275192	‐/1	0.008		0.003560	0.0004
8	*GNPTG*	c.813G > A	p.Thr271Thr	Exon 10	rs377647926	‐/18	0.14		unknown	0.00002
9	*NAGPA*	c.333 A > G	p.Gly111Gly	Exon 2	rs2972272	41/19	0.789	229/1708	0.8222	0.6981
10	*NAGPA*	c. 1485C > T	p.Asn495Asn	Exon 10	rs887854	42/22	0.828		0.8177	0.6967
Noncoding variants										
11	*GNPTG*	‐4 C > T	—	5′UTR	rs554707396	‐/1	0.008		0.0002703	0.00006
12	*NAGPA*	c.1174 + 53C > A	—	intron 7	rs2937112	22/26	0.547		Unknown (0.5730 in 1000 K)	0.2792

**TABLE 2:** Pathogenicity prediction of the variants observed in three genes for stuttering using various bioinformatics tools

S. No.	Location	Nucleotide change	dbSNP ID	General		Functional		Conservation		PolyPhen‐2	I Mutant v2.0	Consurf Score	VarSome (ACMG guidelines)	Pathogenic assertion	ACMG attributes
				DANN	Mutation taster	SIFT	Provean	LRT	Mutation Assessor						
Missense variants															
1	*GNPTAB*	c.3598G > A	rs137853825	0.9982	Disease causing	D	Damaging	Deleterious	Low	PD	Decreased stability	9	Benign	Benign	BS1, BS2 PP2, PP3,PP5
2	*GNPTG*	c.802A > C	rs759796840	0.9696	Disease causing	D	Neutral	Deleterious	Medium	PD	Decreased stability	8	Likely Pathogenic	VUS	PS2, PM2, PP2, PP3
3	*NAGPA*	c.131G > C	rs374266430	0.9819	Polymorphism	D	Damaging	Neutral	Medium	PD	Decreased stability	4	Likely benign	Likely benign	BS1, BP1, BP4
4	*NAGPA*	c.139C > T	rs371054576	0.8056	Polymorphism	T	Neutral	Neutral	Neutral	B	Decreased stability	1	Likely benign	Likely benign	BS1, BP1, BP4
5	*NAGPA*	c.1394 C > T	rs7188856	0.3973	Polymorphism automatic	T	Neutral	Neutral	Low	PD	Decreased stability	1	Benign	Benign	BA1, BP1, BP4, BP6
Synonymous variants															
6	*GNPTAB*	c.1932A > G	rs10778148	0.4236	—	—	—	—	—	—	—	—	Benign	Benign	BA1, BP4, BP6, BP7
7	*GNPTG*	c.702 T > C	rs532275192	0.3353	—	—	—	—	—	—	—	—	Likely benign	Likely benign	PM2, BP4, BP7
8	*GNPTG*	c.813G > A	rs377647926	0.4781	—	—	—	—	—	—	—	—	VUS	VUS	PM2, BP4
9	*NAGPA*	c.333 G > A	rs2972272	0.5259	—	—	—	—	—	—	—	—	Benign	Benign	BA1, BP4
10	*NAGPA*	c. 1485C > T	rs887854	0.7803	—	—	—	—	—	—	—	—	Benign	Benign	BA1, BP4, BP6, BP7
Noncoding variants															
11	*GNPTG*	‐4 C > T	rs554707396	0.9074	—	—	—	—	—	—	—	—	VUS	VUS	PM2, BP4
12	*NAGPA*	c.1174 + 53C > A	rs2937112	0.7424	—	—	—	—	—	—	—	—	VUS	VUS	PM2, BP4

*Note*: DANN score 1: most damaging; D: damaging; T: tolerant; PD: possibly damaging; B: benign; VUS: variant with uncertain significance.

The number of variants observed in *NAGPA* (n = 6) was greater than that observed in *GNPTG* (n = 4) and *GNPTAB* (n = 2) genes. Among the variants identified, those missense variants with high conservation scores and pathogenic predictions in both SIFT and Polyphen were examined further in segregation analysis. Three such missense variants, p. Glu1200Lys in *GNPTAB*, p. Ile268leu in *GNPTG*, and p. Arg44Pro in *NAGPA* gene, were observed in three unrelated probands.

The other two missense variants (p. Leu47Phe and p. Thr465Ile) in *NAGPA*, were observed to have a higher minor allele frequency (MAF) in the gnomAD exome database favoring common variant and likely benign status. Further, these variants showed a low conservation score with benign predictions either in SIFT or Polyphen2. Hence, segregation analysis and genotype‐phenotype correlations were performed only for the three missense variants with a high conservation score and low MAF. Although the p. Glu1200Lys variant in the *GNPTAB* gene has high MAF it was still considered for segregation analysis since it has been previously reported.^18^

### 2.2 Segregation analysis and genotype‐phenotype correlations

#### 2.2.1 Family STU 29

The 16‐year‐old proband with stuttering was ascertained from a government boy's higher secondary school in Salem, Tamil Nadu. He was born to nonconsanguineous parents and had no complications during his birth. His age at onset of stuttering was reported to be 3 years and he was right‐handed. Severity assessment rated his stuttering as severe with an excess of prolongations, blocks, and difficulties in initial syllables. Secondary behaviors include eye blinking, stiffness of the body, the tension in the neck, and avoidance of eye contact. There was a situational increase in the stuttering such as in a classroom while speaking with teachers, superiors, or the opposite sex and when excited or afraid. Both the proband's father and his elder brother had stuttering that could be rated as moderate.

**FIGURE 3:**
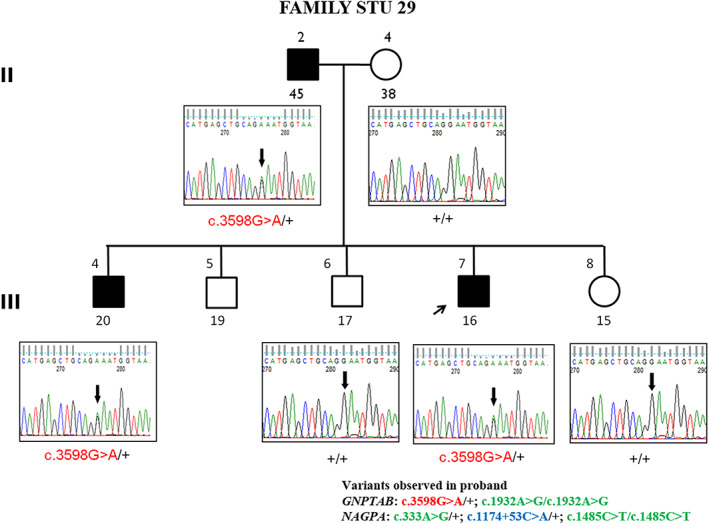
Partial chromatograms of the p.Glu1200Lys mutation (*GNPTAB*) segregating in a family with stuttering

Mutational analysis in the proband identified a heterozygous missense variant that is in a conserved position p. Glu1200Lys in exon 19 of the *GNPTAB* gene. On extending, the analysis to their family members there was cosegregation of the variant with the affected status in a dominant pattern (Figure 3).

Furthermore, since this variant was predicted as pathogenic in previous studies it was screened in an additional subset of 26 individuals with severe stuttering. Three of them had this lysine variant resulting in an allele frequency of 2% (4/180), which is similar to that observed in the gnomAD database (2.1%). This variant's classification according to ACMG is benign (Table 2).

#### 2.2.2 Family STU 63

The proband is a 24‐year‐old male who had normal speech until 9 years. Severity assessment rated his stuttering as mild with repetitions, blocks and had eye closure during speech. The proband was born to nonconsanguineous parents without any complications during birth. He was right‐handed with good academic performance.

**FIGURE 4:**
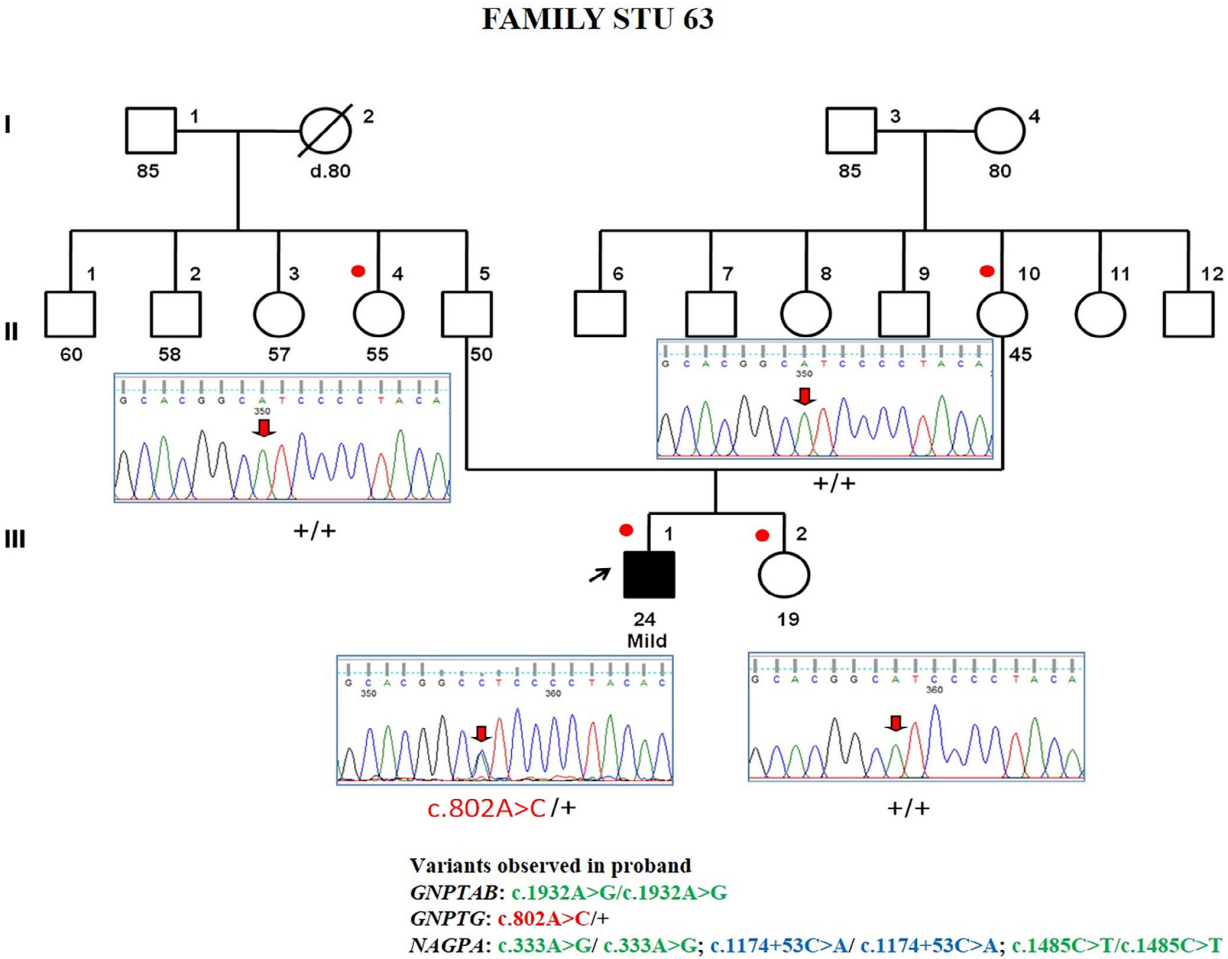
Partial chromatogram of c.802A > C (p.Ile268Leu) variation in *GNPTG* gene

Mutation analysis of the proband identified a heterozygous missense variant p. Ile268Leu in exon 10 of the *GNPTG* gene. On extending the analysis to his family members, his unaffected father, mother and sister did not show this variation. Since this is not present in either of the parent but only observed in the proband, it is identified as a de novo variant (Figure 4). There were no other synonymous variants in the sequenced region to check for the consistency of the paternity. Hence, we typed for Rh blood group phenotype that was found to be consistent with paternity.

As per the ACMG guidelines the variant is classified as “likely pathogenic”. Considering the nonconsanguineous pedigree, and confirmed de novo status of the variant in the affected individual, the variant in *GNPTG* likely favors the gene‐disease relationship with a dominant mode of action.

### 2.3 Impact of the de novo missense variant (c.802A > C/+ in *GNPTG*)

The importance of heterozygous allele in *GNPTG* gene and its relevance in dysfluency disorder, as opposed to the reported recessive mucolipidosis III phenotype due to homozygous truncation of the same *GNPTG* gene was investigated. To study the impact of this de novo heterozygous variant (c.802A > C/+), mRNA expression profile and lysosomal enzyme assay along with mucolipidosis screening test were performed. All the family members including the affected proband, unaffected father, mother, and sister, were screened for mucolipidosis and found negative for the test. The activity of the enzymes studied in plasma was found to be well within the normal range (Table 3).

**TABLE 3:** Lysosomal enzyme study in the plasma of a stuttering family

Family STU 63	Genotype of *GNPTG* gene	Lysosomal enzymes		
		Arylsulfatase A (normal range 30‐268 nmol/h/mg protein)	Hexosaminidase A (normal range 90‐385 nmol/h/mg protein)	Β‐galactosidase (normal range 470‐2500 nmol/h/mg protein)
STU 63‐1 (proband)	c.802A > C/+	32.6	106.9	581.6
STU 63‐2 (father) unaffected	+/+	31.9	108.1	489.7
STU 63‐3 (mother) unaffected	+/+	38.2	113.1	631.9
STU 63‐4 (sister) unaffected	+/+	33.6	116.1	506.3

**FIGURE 5:**
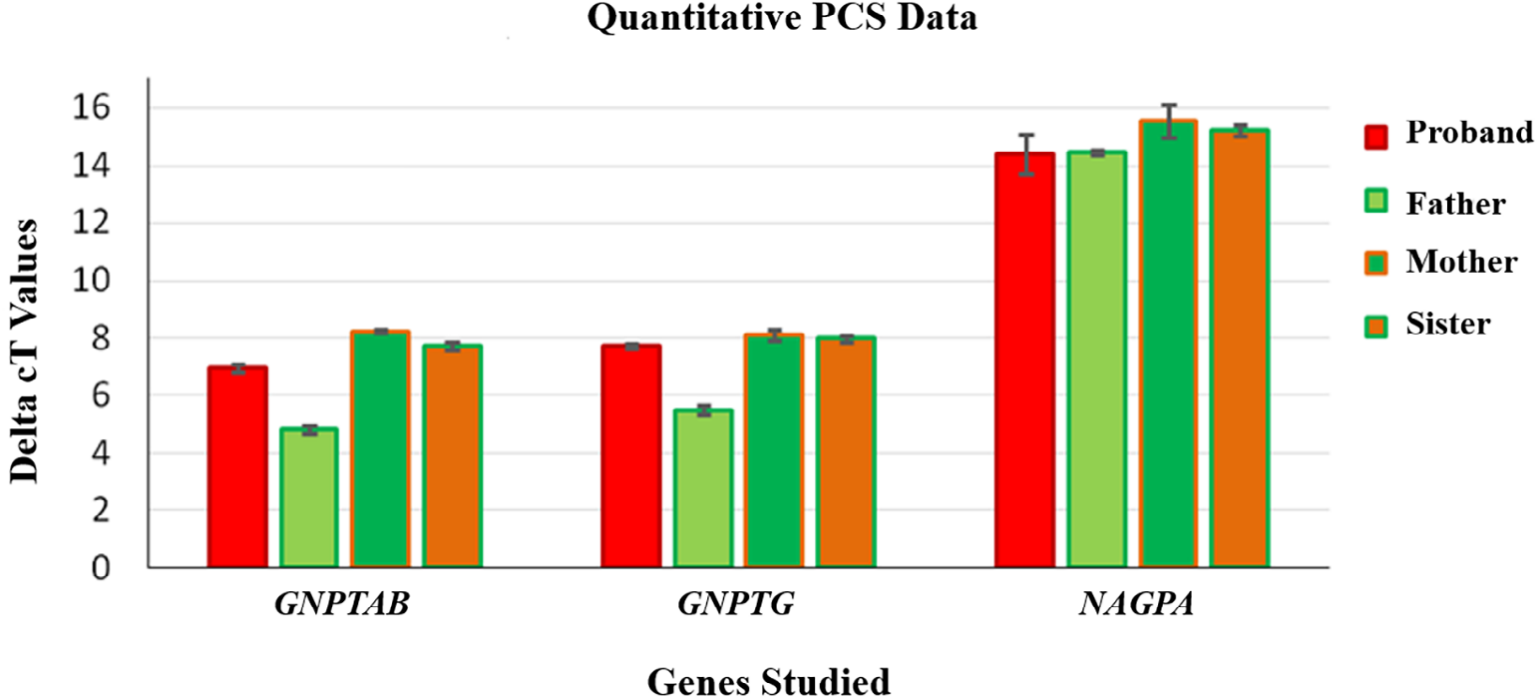
The relative levels of GNPTG, GNPTAB, and NAGPA mRNA expression in triplicates were determined in WBC from blood sample of stuttering patients by real‐time PCR normalized to β‐actin *ACTB* expression. Data indicate ∆Ct values ±SD

To quantify mRNA, the ΔCt values (ΔCt = Ct target—Ct reference) obtained were normalized to the housekeeping β‐actin gene *ACTB* for each of the target genes studied (*GNPTAB*, *GNPTG*, and *NAGPA*) and is shown in the plot (Figure 5). The data suggest that there is variability within the controls (father, mother, and sister) and there is no obvious difference between the proband and the internal control group.

The 2^‐ΔΔCt calculations were performed to check if the control data can be pooled. The ΔΔCt value was obtained by subtracting the ΔCt of a proband with ΔCt of control (ΔΔCt = ΔCt test sample—ΔCt control). Because of the variability, and small sample size, 2^‐ΔΔCt calculation, and statistical significance testing were not feasible. However, the data suggest that the expression of *GNPTG*, as well as *GNPTAB* and *NAGPA* genes in the proband, are within the range of the controls.

#### 2.3.1 Family STU 34

The 15‐year‐old male proband with stuttering born to nonconsanguineous parents was ascertained from a National high school in Salem, Tamil Nadu, and had no complications during his birth. The age at onset of stuttering was unknown and reported as sudden and is right‐handed with good academic performance. Severity assessment rated him as severe with prolongations, blocks, irregular breathing, and with difficulties in initial syllables. Secondary behaviors were mild. There was a situational increase in the stuttering in a classroom while speaking with teachers, etc.

**FIGURE 6:**
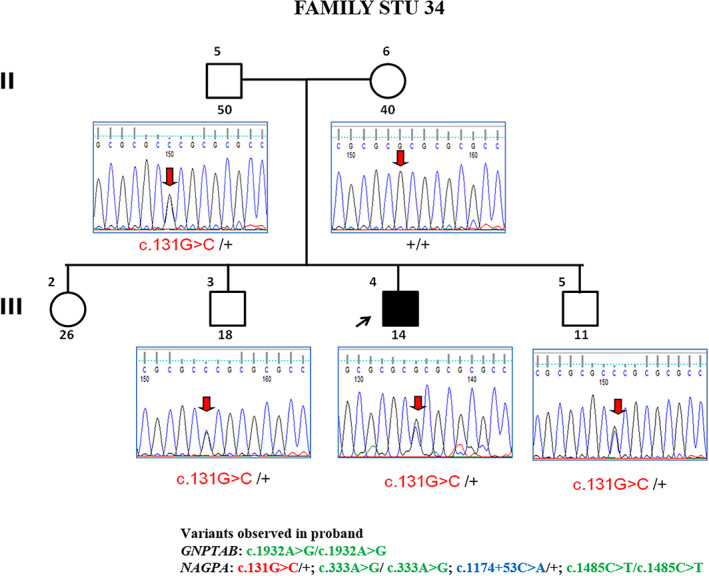
Partial chromatogram of c.131G > C (p.Arg44Pro) mutation in *NAGPA* gene

Mutation analysis identified a heterozygous missense variant p. Arg44Pro in exon 2 of the *NAGPA* gene. Extended testing and analysis revealed the presence of this variant in his unaffected father and his two unaffected brothers (Figure 6). Also, ACMG classifies this variant as benign.

Table 4 lists a comprehensive variant profile observed in the three putative stuttering implicated genes in all the three probands presented above. The genes are known to be functionally related and belong to the lysosomal targeting pathway. On examining the variant profiles of these three probands (STU 29, STU 63, and STU 34) there was a co‐occurrence of synonymous and noncoding variants with unknown clinical significance.

**TABLE 4:** Variant profile of the probands in the three putative genes for stuttering

SAMPLE	*GNPTAB*		*GNPTG*		*NAGPA*	
	HET	HOMO	HET	HOMO	HET	HOMO
STU 29	c.3598G > A (E19)	c.1932A > G (E 13)			c.333 A > G (E2)	c. 1485C > T (E10)
					c.1174 + 53C > A (I7)	
STU 63		c.1932A > G (E 13)	c.802A > C (E10)			c.333 A > G (E2)
						c. 1485C > T (E10)
						c.1174 + 53C > A (I 7)
STU 34		c.1932A > G (E 13)			c.131G > C(E2)	c.333 A > G (E2)
					c.1174 + 53C > A (I7)	c. 1485C > T (E10)

Bioinformatic analysis of the alignment of the secondary structure of mutated GNPTAB protein (p. Glu1200Lys) with the native protein identified loss of helix and addition of turn near the mutated region (Supplementary Figure A4a). A similar analysis for GNPTG protein (p. Ile268Leu) showed that the mutation did not affect the secondary structure (Figure A4b).

Segregation analysis revealed that only one variant, p. Glu1200Lys in *GNPTAB* gene, cosegregated with the affected individual (Table 5) while p. Ile268Leu in *GNPTG* gene was found to be de novo. Since the *GNPTAB* variant is benign, the allele frequency of all likely causative variants was 0.8% (1/128) in the stuttering families studied.

**TABLE 5:** Segregation pattern and genotype‐phenotype correlation of variants identified in the putative genes for stuttering among three probands

Code	Individual	Age	Sex	Phenotype	Gene	Genotype	Remarks
STU‐29							
II‐2	Father	45	M	Affected	*GNPTAB*	c.3598G > A/+	Cosegregation of the variant suggests a dominant inheritance pattern Familial nonconsanguineous
II‐4	Mother	38	F	Unaffected		+/+	
III‐4	Brother	20	M	Affected		c.3598G > A/+	
III‐6	Sister	17	F	Unaffected		+/+	
III‐7	Proband	16	M	Affected		c.3598G > A/+	
III‐8	Younger brother	15	M	Unaffected		+/+	
STU‐63							
II‐5	Father	50	M	Unaffected	*GNPTG*	+/+	de novo variation Sporadic nonconsanguineous
II‐10	Mother	45	F	Unaffected		+/+	
III‐1	Proband	24	M	Affected		c.802A > C/+	
III‐2	Sister	19	F	Unaffected		+/+	
STU‐34							
II‐5	Father	50	M	Unaffected	*NAGPA*	c.131G > C/+	The variation does not cosegregate with affected status Sporadic nonconsanguineous
II‐6	Mother	40	F	Unaffected		+/+	
III‐3	Brother	18	M	Unaffected		c.131G > C/+	
III‐4	Proband	14	M	Affected		c.131G > C/+	
III‐5	Younger brother	11	M	Unaffected		DNA unavailable	

## 3 DISCUSSION

Speech is a robust faculty that serves most people in the face of various challenges. Nevertheless, developmental stuttering is a complex phenotype with overt diversity in terms of both genetic and deterministic risk factors.

### 3.1 Mutational analysis of implicated genes in stuttering

The present study will be the first research report on the Indian stuttering cohort to be investigated on the recurrence and implication of three functionally related lysosomal enzyme targeting pathway genes, *GNPTAB*, *GNPTG*, and *NAGPA*. We have screened for the complete coding sequence of 12 specific exons where the variants have been previously reported. Our analysis focused not only on the previously reported variants but also on any other variants that occurred in these 12 exons studied.

A meta‐analysis from worldwide reports on unrelated PWS has identified 81 rare nonsynonymous coding variants, in either of the three putative genes, yielding a frequency of 16% (164/1013).^25^ While we have observed four nonsynonymous coding variants that accounted for 6% (4/64) in the present study. Among the 12 variants identified, 5 of them (p. Glu1200Lys and p. Thr644Thr in *GNPTAB* and p. Arg44pro, p. Gly111Gly and p. Asn495Asn in *NAGPA*) were previously reported in stuttering populations. One variant (p. Pro234Pro in *GNPTG*) was reported in mucolipidosis III. However, the remaining six variants (p. Ile268Leu, p. Thr271Thr, c.‐4 C > T in *GNPTG* and p. Leu47Phe, p. Thr465Ile, c.1174 + 53C > A in *NAGPA*) were observed in the gnomAD public database. Three of the conserved nonsynonymous variants (p. Glu1200Lys in *GNPTAB*, p. Ile268leu in *GNPTG*, and p. Arg44Pro in *NAGPA* gene) that were considered for segregation analysis are discussed individually.

### 3.2 STU 29 family with c.3598G > A variant in *GNPTAB* gene

The fact that the highest linkage scores were obtained for the variant c.3598G > A in *GNPTAB* combined with the lack of another plausible genetic variant within the linkage interval highlighted that this may have had an increased risk of stuttering when this variant is present in either one or two copies.^18^ Fedyna et al.^26^ reported 4/8 unrelated PWS carried at least one copy of p.Glu1200Lys variant in *GNPTAB* gene and established this as a founder mutation of the Asian population, originating from Pakistan and India.

Recurrence of this lysine variant in heterozygous condition (0.8%) segregating with affected status in our study favors the founder effect in Asians. Although the lysine variant is highly conserved across species (Consurf = 9^28^) with pathogenicity predictions and also in silico prediction of its effect on protein structure was shown to disrupt the helical segment which may be crucial for protein‐protein interaction (Supplementary Figure A4), its high frequency among South Asian ancestry (2.1%) in the gnomAD database is inconsistent with a causal role in stuttering pathology. Eight individuals in the south Asian population also had this homozygous variant indicating that this variant is not lethal, unlike *GNPTAB* homozygous variants observed in mucolipidosis. Hence, this lysine variant is confirmed to be benign in the present study.

In a recent animal model study, 3‐ to 8‐day‐old mice pups were engineered to carry two copies of the lysine mutation (Gnptab^mut/mut^), resulting in significantly longer pauses in their spontaneous vocalizations consistent with some features of human stuttering. This was neither observed in littermates without the mutation (Gnptab^wt/wt^) nor in heterozygous (Gnptab^mut/wt^) littermates.^20^ Based on this mouse model study, the causative role was well established for the homozygous lysine variant in the *GNPTAB* gene, while the heterozygous variant effect was notably similar to wild‐type phenotypically. However, the significance of this variant acting in *trans* with other genetic determinants in other coding and noncoding exons could not be excluded. Further studies in this direction to identify new interacting genes or functionally related gene candidates in common pathways may clarify the causative role of this gene in stuttering.

In complex disorders, the genotype‐phenotype correlation is rarely simple as dominance and recessive patterns for a given gene as described by Mendel. In many cases, multiple alleles contribute to a trait and include a variety of relationships between alleles (allelic interactions) that code for the same trait. Allelic dominance constantly depends on the relative influence of each allele responsible for the phenotype under given environmental conditions. Dominance and recessiveness are not essentially allelic properties but measured with respect to the effects of other alleles at the same locus. Additionally, dominance may change according to the level of organization of the phenotype and its variations highlight the complexity of understanding genetic influences on phenotypes.^27^ Two other *GNPTAB* homozygous mutations p.Ser321Gly and p.Ala455Ser engineered in mice, also displayed vocalization deficits traceable to abnormalities in astrocytes of corpus callosum.^21^

### 3.3 STU 63 family with c.802A > C variant in *GNPTG* gene

This heterozygous variant was found only in the proband, but absent in other family members and hence it is confirmed to be de novo. It was not observed so far in the stuttering population but was reported in the gnomAD database in a heterozygous state with no information on MAF. In general, de novo variants are considered clinically significant due to constraints in natural selection and evolutionary conservation. The majority of genetic etiology studies of severe neurodevelopmental disorders associated with speech impairment were reported to be due to de novo mutations. They may be prime candidates when genetic diseases occur sporadically.^29^ In our study, the role of this de novo variant in stuttering is ostensibly supported by a high conservation score. Investigation of the recurrence of this mutation is warranted in unrelated PWS that may provide further evidence of the GNPTG gene to neurobiological underpinnings of stuttering. Intriguingly, a recent study by Benito‐Aragón et al.^30^ had elucidated that GNPTG—a gene involved in the M6P lysosomal targeting pathways—was significantly colocalized with the stuttering cortical network based on functional connectivity MRI and graph theory. This study had utilized a spatial similarity analysis approach that elucidated the topology of the stuttering cortical network by intersecting with genetic expression levels of previously reported genes for stuttering from the protein‐coding transcriptome data of the Allen Human Brain Atlas.

Most of the mutations hitherto reported in stuttering are heterozygous. Hence, a question arises: Are they dominant in stuttering in contrast to the recessive state observed in all mucolipidosis? Hence, to study the impact of the de novo heterozygous missense variant identified in *GNPTG*, (a) quantification of mRNA by RT‐PCR, (b) activity of lysosomal enzymes in plasma, (c) mucolipidosis screening was carried out between the affected and unaffected members of the family. Does this heterozygous condition have any impact on the expression and activity of the GNPTG enzyme? We also quantified two other genes *GNPTAB* and *NAGPA*, involved in M6P formation to examine whether the defect in the *GNPTG* gene affects the expression of other components.^31^ Further *GNPTG* and *GNPTAB* genes encode different subunits of the same enzyme and there is a possible feedback regulation mechanism between them.^32^

If the variant affects the targeting function, the lysosomal enzymes will not be targeted to lysosomes but will be secreted in plasma. Thus, the enzyme deficiency can be demonstrated by elevated lysosomal enzyme activity in plasma.^33^ Nevertheless, in our study the activity of GNPTG enzymes was not elevated, indicating that it might be successfully targeted to lysosomes. Also, the proband with stuttering did not have any symptoms of mucolipidosis and tested negative. We propose that, since the variation observed is in heterozygous condition, either the normal copy is sufficient or this variant does not affect the function of the enzyme. Similarly, there was no fold change in the mRNA level of the three genes between the affected (proband) and unaffected members (father, mother, and sister) of the family.

### 3.4 STU 34 family with c.131G > C variant in *NAGPA* gene

Only one isolated case of European descent has been reported to have this variant among the stuttering population studied.^25^ Observation of this variant in both affected and unaffected family members in our study suggests that this variant is less likely to have a causal effect. This may also be explained by incomplete penetrance that may fail to show any symptoms in some or could be due to phenocopies in affected members who may not be real carriers of variant but tend to display stuttering under environmental effects.^18^ Also, variants observed in normal individuals may have caused stuttering but left un‐informative owing to early recovery^34^; nevertheless, in the present study, we did not find any such recovery in this family.

### 3.5 Role of synonymous and noncoding variations

Overall, five synonymous variants and two noncoding variants were observed in our cohort. Synonymous mutations are often considered silent mutations due to the degeneracy of genetic code. However, they may have important consequences and are now recognized to be crucial in influencing gene expression, conformation, and cellular function.^35^ Complex disorders often tend to have multiple mutations. A mutation may not be detrimental individually but the joint effect of multiple variants in the same gene or different genes can contribute to a disorder but predictions are limited to a single variant.^36^

Although our study has identified some of the reported variants, the perplexing question of causal role or the pathogenicity of a variant in the heterozygous state in stuttering or dysfluency disorder is still debatable. The process of speech‐producing mechanism is believed to be highly heterogeneous due to its overlapping phenotypes with various genetic conditions and underlying etiology. Next generation sequencing based assays in individuals with stuttering will certainly throw light on complex disorders in terms of understanding the genetic heterogeneity, mutation burden in genes associated with speech‐brain pathways.

## 4 CONCLUSION

Decades of research from multiple groups have tied stuttering to be heritable, with evidence of genetic variants providing insight on detectable changes in the brain, which is an advancement. Mutation screening of the three stuttering implicated genes (*GNPTAB*, *GNPTG*, and *NAGPA*) among 64 PWS, resulted in recurrence of some of the previously reported variants. The only causative variant that could be attributed to stuttering was the de novo variant found in *GNPTG*, yet the causative role in the disorder, as opposed to the recessive nature of these genes, remains elusive. While not delimiting our observations, we emphasize the need for similar studies to evaluate the heterozygous nature of variants in genes of the lysosomal pathway. The involvement of more stuttering genes is predicted and can be well addressed using NGS technology.

## 5 METHODS

### 5.1 Ethical Review and Informed Consent

This study was approved by the Institutional Ethical Committee of the Institute of Basic Medical Sciences, University of Madras, and informed consent was obtained from all participants (Approval No: UM/IHEC/10‐2017 ‐I).

### 5.2 Research participants and Clinical evaluation

Sixty‐four probands were recruited from various schools, hospitals, and speech therapy clinics and clinically diagnosed for stuttering by speech pathologists using Stuttering Severity Instrument 3.^37^ It measures stuttering severity using three parameters: (a) frequency of stuttering, expressed as a percentage of words stuttered, (b) duration which is the average of the three longest stuttering moments, and (c) recognizable physical concomitants, culminating in a single total overall score.^38^ A structured interview using a questionnaire was conducted to elicit demogenetic details (Supplementary Table A1). More details on the recruitment of the probands are given in our previous paper.^4^

An additional subset of 26 severe stuttering adults was recruited from a stuttering self‐help group annual conference. This subset was utilized interim to answer a specific question during the progress of the work.

Eight milliliters of blood sample were collected from probands by venipuncture into labeled EDTA coated vacutainers (Beckon and Dickinson Co.). Genomic DNA was isolated using the phenol‐chloroform extraction method.^39^ The family members of the probands were included in the study.

### 5.3 Mutational analysis of *GNPTAB*, *GNPTG*, and *NAGPA* genes

The 12 specific exons spanning across the three genes namely, *GNPTAB*, *GNPTG*, and *NAGPA* implicated in stuttering were screened (Figure 1). Our analysis was focused to investigate on the known and any novel variants that occurred in these 12 exons studied. Primer sequences were adapted from Kang et al. (2010)after improvising using NCBI's Primer‐BLAST^40^ the sequence coverage of exon 10 of the *NAGPA* gene. As a cost‐effective approach, we selected these 12 exons to observe the recurrence of mutation in our ethnic population that is unexplored to this date.

The amplified PCR products were purified by the FavorPrep PCR purification kit (FAVORGEN, Taiwan). The amplicons were sequenced using ABI Prism Big‐Dye Terminator 3.1 cycle sequence reaction kit on ABI 3730XL automated sequencer (Applied Biosystems). Chromatograms were analyzed using NCBI nucleotide BLAST^41^ and UCSC genome browser BLAT^42^ (GRCh37/hg19 build).

Variants identified were predicted using VarSome^43^ tools include various predictors like DANN, mutation taster, likelihood ratio test [LRT], mutation assessor, SIFT, Provean, etc.) and Polyphen tool, to deduce the pathogenicity. It was classified according to ACMG guidelines along with the ACMG attributes. The guidelines describe the process of classifying variants into five categories as “pathogenic,” “likely pathogenic,” “uncertain significance,” “likely benign,” and “benign,” based on the evidence from computational data, population data, functional data, and segregation data.^44^ Cosegregation of the pathogenic variants among the family members was also evaluated. The novelty and frequency of the variants were compared with the gnomAD database that served as a control. They represent the general population and few individuals may likely be affected or recovered from the disorder of interest.

### 5.4 Effects of p. Glu1200Lys and p. Iso268Leu amino acid change in GNPTAB and GNPTG protein, respectively

Both the native and mutated protein of GNPTAB and GNPTG were subjected to pair‐wise alignment using the Geneious Pro version 6.1.2.^45^ The pair‐wise alignment was carried out by MAFFT alignment. Default parameters were set to assess and predict the effect of SNP identified in this study.

### 5.5 Impact of a de novo variant c.802A > C/+ (p. Ile268Leu) by quantifying gene expression

To study the impact of a de novo heterozygous variant (c.802A > C/+) in *GNPTG* gene identified in one family (STU 63), mRNA expression profile, and lysosomal enzyme study was performed along with mucolipidosis screening test. The proband was the only individual affected with stuttering, while his father, mother, and sister served as normal speaking controls. The plasma collected from fresh blood (5 mL) was used to study the enzyme activity. RNA was isolated using mirVana miRNA isolation kit (Invitrogen) as per the manufacturer's protocol and checked for integrity and purity.

A two‐step qRT‐PCR was used to measure the transcript levels of the mRNAs of interest.

1. From 500 ng of total RNA, cDNA was synthesized using the RevertAid First Strand cDNA Synthesis Kit (Thermo Scientific) according to the manufacturer's instruction in ABI GeneAmp 9700 PCR System. A reverse transcription reaction mix of 20 μL was prepared and was loaded on to ABI GeneAmp 9700 PCR system.
2. For quantifying gene expression, real‐time quantitative PCR was performed on QuantStudio3 Real‐Time PCR System using GoTaq DNA polymerase (Promega) in the presence of SYBR Green. The primers specific for the transcripts of *GNPTAB*, *GNPTG*, and *NAGPA* (Supplementary Table A2) were designed with the aid of IDT software^46^ and checked for specificity with NCBI's primer‐blast. The annealing temperature of the primers was optimized using temperature gradient PCR. Reaction mix for the samples under investigation along with NTC (no template control) was prepared in triplicates for *GNPTAB*, *GNPTG*, *NAGPA*, and *ACTB* genes. The reaction mix was loaded onto a 96 well plate and sealed with MicroAmp Optical Adhesive Film (Applied Biosystems).

Ct value (cycle threshold) is the number of PCR cycles required to achieve a given level of fluorescence. Since the Ct value is proportional to the logarithm of the initial amount of the target, the relative concentration of one target with another is reflected as a difference in cycle number (ΔCt) that is necessary to achieve an equivalent level of fluorescence. The expression levels of *GNPTAB*, *GNPTG*, and *NAGPA* were measured by relative quantification using the ΔCt method with β‐actin gene *ACTB* as endogenous control.

Delta Ct values were normalized to the housekeeping β‐actin gene *ACTB* for each of the target genes (*GNPTAB*, *GNPTG*, and *NAGPA*). Statistical analysis was performed by averaging the control Delta Ct values and comparing it with the respective gene expression.

Lysosomal targeting of proteins was studied using a specific substrate for arylsulfatase A (ASA), hexosaminidase A, and β galactosidase enzymes, with plasma samples from stuttering proband and controls. All members in the family were also evaluated for mucolipidosis phenotype using a rapid colorimetric screening method. It is a simple chemical test where the synthetic substrate p‐nitrocatechol sulfate gets hydrolyzed in presence of ASA when excessively present in the plasma and catalyzes to form excess pNC. It gives dark brown color in an alkaline solution which is visible to the naked eye.^33^

## CONFLICT OF INTEREST

The authors declare no actual or potential conflicts of interest to disclose.

## AUTHOR CONTRIBUTIONS

**C. R. Srikumari Srisailapathy**: Conceptualization; funding acquisition; project administration; resources; supervision; writing‐review and editing. **Gunasekaran Nandhini Devi**: Conceptualization; data curation; formal analysis; investigation; methodology; project administration; validation; writing‐original draft; writing‐review and editing. **Jayasankaran Chandru**: Validation; writing‐review and editing. **Justin Margret Jeffrey**: Resources; writing‐review and editing. **Krishnamoorthy Mathuravalli**: Methodology.

### PEER REVIEW

The peer review history for this article is available at https://publons.com/publon/10.1002/ggn2.10043.

## Supporting information


**Supplementary Figure A1** Partial chromatograms of *GNPTAB* variants observed in the study
**Supplementary Figure A2**: Partial chromatograms of *GNPTG* variants observed in the study
**Supplementary Figure A3**: Partial chromatograms of variants observed in *NAGPA* gene
**Supplementary Figure A4a**: MAFFT alignment of native and mutated secondary structure of GNPTAB protein using the Geneious Pro version 6.1.2 identified the loss of helix and addition of turn at the site of mutation.
**Supplementary Figure A4b**: MAFFT alignment of native and mutated secondary structure of GNPTG protein using the Geneious Pro version 6.1.2 showing no change in the secondary structure

Supplementary TPR File

## Data Availability

No data were deposited.
