# Supplementary material for: Evaluation of recurrent GNPTAB, GNPTG, and NAGPA variants associated with stuttering
Source: Adv Genet (Hoboken). 2021 May 20;2(2):e10043. doi: 10.1002/ggn2.10043 (PMC9744500; doi:10.1002/ggn2.10043)
Supplement: Supplementary file 1 — Supplementary Figure A1 Partial chromatograms of GNPTAB variants observed in the study Supplementary Figure A2: Partial chromatograms of GNPTG variants observed in the study Supplementary Figure A3: Partial chromatograms of variants observed in NAGPA gene Supplementary Figure A4a: MAFFT alignment of native and mutated secondary structure of GNPTAB protein using the Geneious Pro version 6.1.2 identified the loss of helix and addition of turn at the site of mutation. Supplementary Figure A4b: MAFFT alignment of native and mutated secondary structure of GNPTG protein using the Geneious Pro version 6.1.2 showing no change in the secondary structure [file GGN2-2-e10043-s001.pdf]

Supplementary Figures and Tables

Evaluation of recurrent *GNPTAB*, *GNPTG* and *NAGPA* variants associated with stuttering

Nandhini Devi G[1], Chandru J[1,2], Jeffrey JM[1], Mathuravalli K[1], Srikumari Srisailapathy CR[1]*

1.Department of Genetics, Post Graduate Institute of Basic Medical Sciences, University of Madras, Taramani campus, Chennai 600 113, India.
2. LifeBytes India Pvt. Ltd., Bengaluru, Karnataka, India
**Corresponding author Email: srikumarinandhini@gmail.com* and crsrikumari@gmail.com

List of Appendices

Supplementary Figures

Supplementary Figure A1: Partial chromatograms of *GNPTAB* variants observed in the study
Supplementary Figure A2: Partial chromatograms of *GNPTG* variants observed in the study
Supplementary Figure A3: Partial chromatograms of variants observed in *NAGPA* gene
Supplementary Figure A4a: MAFFT alignment of native and mutated secondary structure of GNPTAB protein using the Geneious Pro version 6.1.2 identified the loss of helix and addition of turn at the site of mutation.
Supplementary Figure A4b: MAFFT alignment of native and mutated secondary structure of GNPTG protein using the Geneious Pro version 6.1.2 showing no change in the secondary structure

Supplementary Tables

Supplementary Table A1: Demogenetic details of 64 probands with stuttering involved in mutation screening
Supplementary Table A2: Real time nucleotide primer sequences of target (*GNPTAB*, *GNPTG*, *NAGPA*) and endogenous (*β-actin ACTB*) genes

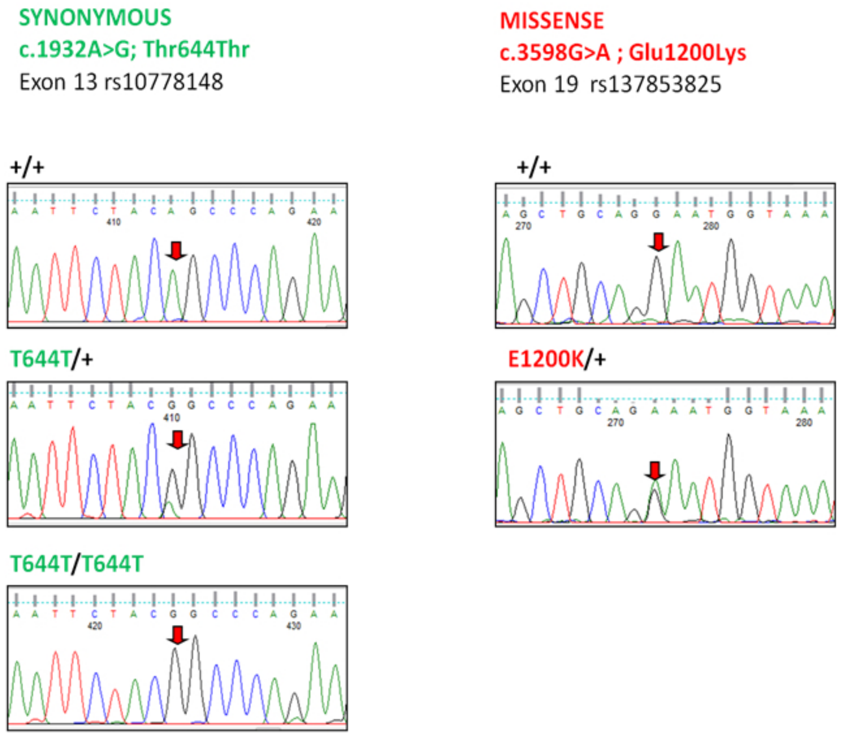

Supplementary Figure A1: Partial chromatograms of *GNPTAB* variants observed in the study

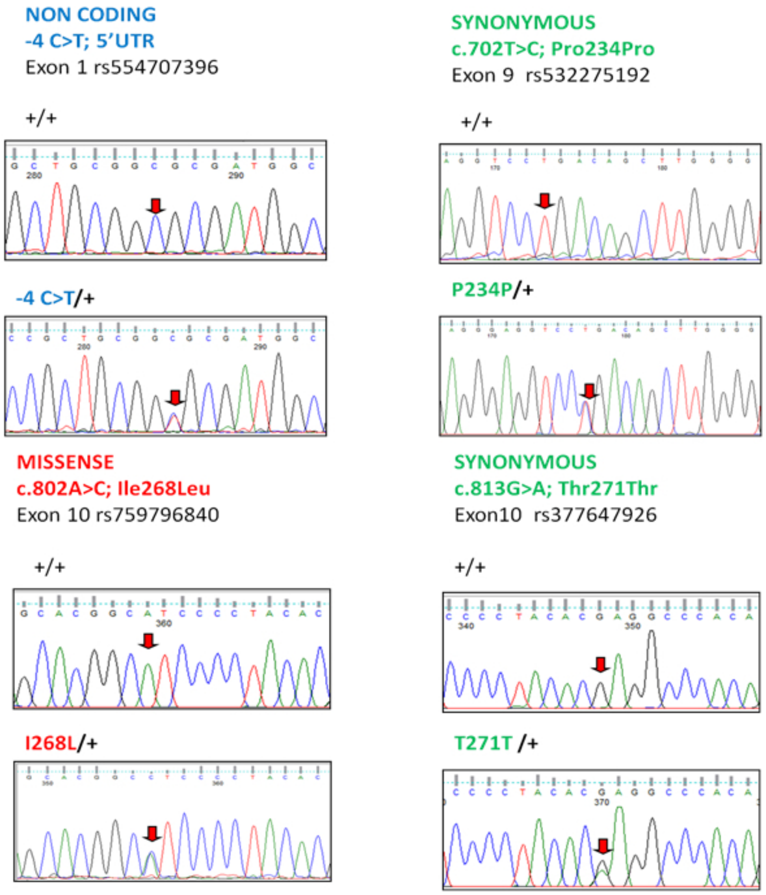

Supplementary Figure A2: Partial chromatograms of *GNPTG* variants observed in the study

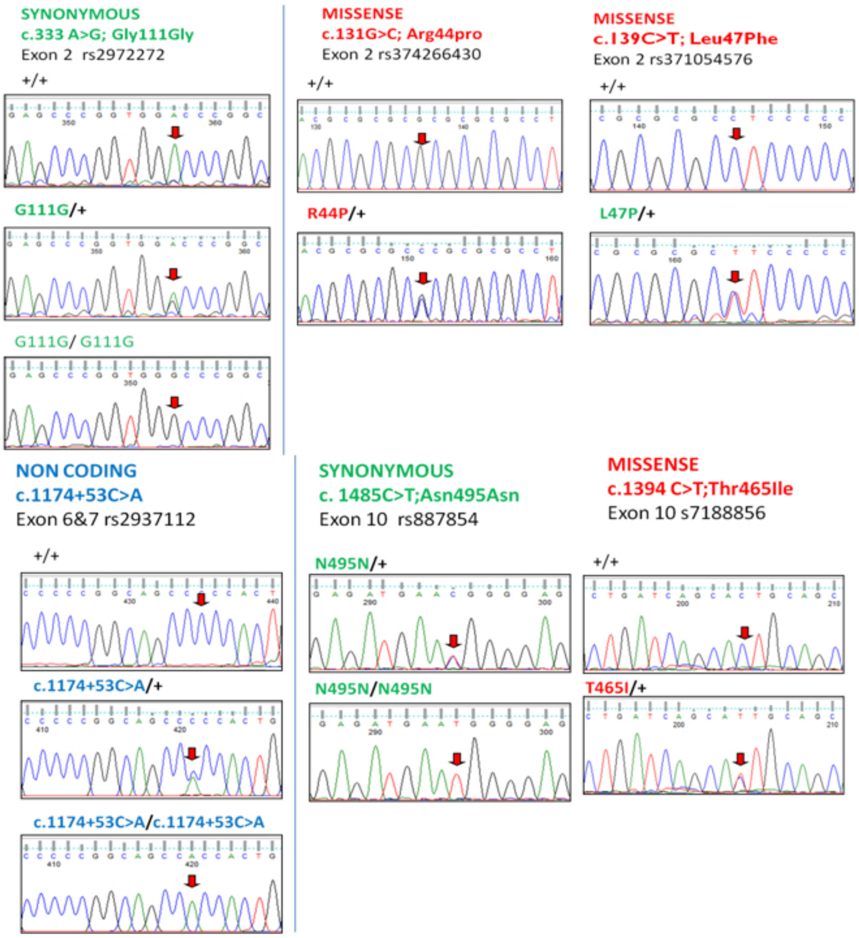

Supplementary Figure A3: Partial chromatograms of variants observed in *NAGPA* gene

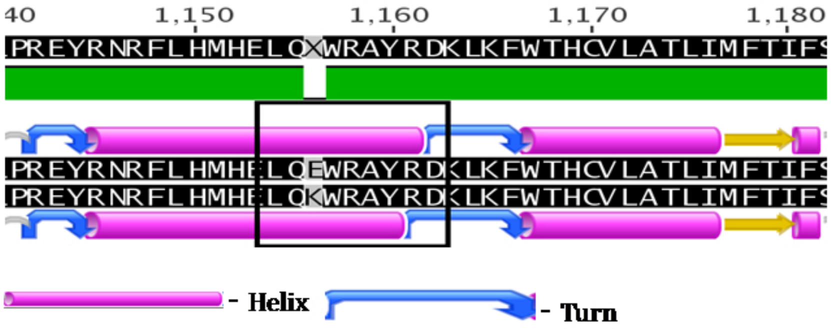

Supplementary Figure A4a: MAFFT alignment of native and mutated secondary structure of GNPTAB protein using the Geneious Pro version 6.1.2 identified the loss of helix and addition of turn at the site of mutation.

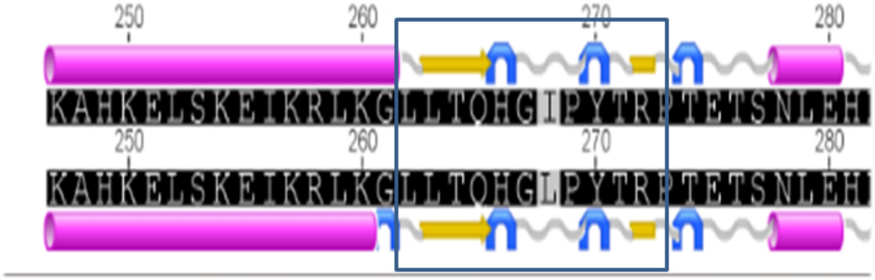

Supplementary Figure A4b: MAFFT alignment of native and mutated secondary structure of GNPTG protein using the Geneious Pro version 6.1.2 showing no change in the secondary structure

CODE	AGE	GENDER	ONSET	SEVERITY	FAMILY HISTORY	CONSANGUINITY
STU 1	20	Male	2	Severe	Familial	NC
STU 2	21	Male	3	Mild	Sporadic	NC
STU 3	13	Male	10	Mild	Familial	NC
STU 4	22	Male	2	Mild	Familial	NC
STU 5	21	Male	15	Severe	Familial	NC
STU 6	27	Male	12	Moderate	Familial	NC
STU 7	15	Male	3	Moderate	Sporadic	NC
STU 8	21	Male	4	Moderate	Familial	First cousins
STU 9	32	Male	5	Severe	Familial	NC
STU 10	24	Male	10	Mild	Sporadic	NC
STU 11	20	Male	3	Mild	Sporadic	NC
STU 12	15	Male	3	Mild	Familial	NC
STU 13	21	Male	3.5	Moderate	Familial	NC
STU 14	22	Male	3	Mild	Familial	NC
STU 15	30	Male	3	Mild	Familial	NC
STU 16	11	Male	6	Moderate	Familial	NC
STU 17	22	Male	-	Mild	Familial	NC
STU 18	11	Male	3.5	Severe	Sporadic	NC
STU 19	10	Male	5	Mild	Familial	NC
STU 20	13	Male	3	Severe	Familial	NC
STU 21	15	Male	3	Severe	Sporadic	NC
STU 22	14	Male	3	Very severe	Familial	NC
STU 23	4	Male	-	Severe	Familial	NC
STU 24	12	Male	3	Severe	Familial	NC
STU 25	16	Male	3	Severe	Familial	DR
STU 26	13	Male	-	Severe	Familial	1 ½ Cousins
STU 27	8	Female	5	Severe	Familial	1 ½ Cousins
STU 28	15	Male	3	Severe	Sporadic	DR
STU 29	16	Male	3	Severe	Familial	DR
STU 30	18	Male	10	Severe	Familial	DR
STU 31	16	Male	3	Severe	Sporadic	DR
STU 32	12	Male	10	Severe	Sporadic	DR
STU 33	12	Male	5	Severe	Familial	Second cousins
STU 34	15	Male	-	Severe	Sporadic	DR
STU 35	15	Male	5	Severe	Sporadic	DR
STU 36	13	Male	3	moderate	Familial	First cousins
STU 37	15	Male	-	Severe	Familial	DR
STU 38	18	Male	-	moderate	Sporadic	NC

STU 39	10	Male	3	moderate	Familial	NC
STU 40	27	Male	3	Severe	familial	DR
STU 41	26	Female	5	Severe	Familial	First cousins
STU 42	20	Female	7	Severe	Familial	NC
STU 43	16	Male	4	Very severe	Sporadic	NC
STU 44	15	Male	-	Very severe	Familial	NC
STU 45	22	Male	6	Moderate	Sporadic	NC
STU 46	30	Male	10	Moderate	Familial	NC
STU 47	9	Male	3	Moderate	Sporadic	First cousins
STU 48	23	Male	Insidious	Moderate	Sporadic	NC
STU 49	14	Male	3	moderate	Familial	NC
STU 50	15	Male	3	Moderate	Familial	NC
STU 51	16	Male	2	Moderate	Sporadic	NC
STU 52	13	Male	5	Severe	Familial	NC
STU 53	13	Male	5	Very severe	Sporadic	NC
STU 54	13	Female	10	Severe	Familial	NC
STU 55	26	Male	9	Very severe	Sporadic	DR
STU 56	21	Male	6	Very severe	Familial	NC
STU 57	12	Male	2	Moderate	Familial	NC
STU 58	10	Male	4	Severe	Familial	NC
STU 59	14	Male	3	Severe	Sporadic	NC
STU 60	26	Female	-	Mild	Familial	C
STU 61	30	Male	11	Moderate	Familial	NC
STU 62	52	Male	2	Severe	Familial	NC
STU 63	24	Male	9	Mild	Sporadic	NC
STU 64	20	Male	10	Moderate	Familial	C

Supplementary Table A1 : Demogenetic details of 64 probands with stuttering involved in mutation screening

GENE	Forward primer (5'-3')	Reverse primer (5'-3')
GNPTAB	TGGCTCGCTGATAAGTTCTG	GTGAGTCTGGTTTGGGAGAAG
GNPTG	CCTTGCTAGTGTACCCAACC	GGTCTTTAAGTAGCCAGCATCC
NAGPA	CTCCAGAGTAAAGCAGTGTCTC	AGGAGCAAGGACAGGTTTG
β -actin	ACCTTCTACAATGAGCTGCG	CCTGGATAGCAACGTACATGG

Supplementary Table A2: Real time nucleotide primer sequences of target (*GNPTAB*, *GNPTG*, *NAGPA*) and endogenous (β-*actin*) genes
