# Supplementary material for: Evaluation of recurrent GNPTAB, GNPTG, and NAGPA variants associated with stuttering
Source: Adv Genet (Hoboken). 2021 May 20;2(2):e10043. doi: 10.1002/ggn2.10043 (PMC9744500; doi:10.1002/ggn2.10043)
Supplement: Supplementary file 2 — Supplementary TPR File [file GGN2-2-e10043-s002.pdf]

ADVANCED GENETICS

Open Access

Evaluation of recurrent *GNPTAB*, *GNPTG* and *NAGPA* variants associated with stuttering

Nandhini Devi G[1], Chandru J[1,2], Jeffrey JM[1], Mathuravalli K[1], Srikumari Srisailapathy CR[1]*

*Corresponding

Review timeline:

Date Submitted: 07-Oct-2020
Editorial Decision: 04-Nov-2020 Major Revision
Revision Received: 13-Jan-2021
Editorial Decision: 10-Feb-2021 Major Revision
Revision Received: 21-Apr-2021
Editorial Decision: 21-Apr-2021 Accept in principle with textual revisions
Revision Received: 02-May-2021
Accepted: 03-May-2021

Editor: Myles Axton

Initial Editorial Evaluation	12-Oct-2020

Summary

64 probands with stuttering (PWS) were Sanger sequenced across 12 exons of GNPTAB, GNPTG and NAGPA. 12 missense variants were found, 5 of which were previously observed in stuttering cases, 3 likely deleterious variants were found heterozygous. GNPTAB c.3598G>A (p.Glu1200Lys, rs137853825, gnomAD 12-102147154-C-T (GRCh37)) cosegregated with 3 affected and not 3 unaffected in pedigree STU29, is probably a founder mutation in South Asians (South Asian ExAC 0.02, total ExAC 0.0035) and in 64+26 PWS appears to have 2.2% allele frequency in PWS. That would mean that the OR~1 (frequency in PWS: frequency in South Asian population or that there is no effect of GNPTAB variants on PWS).
Although the ExAC data (from 7.4m small variants) are similar, it would be better to cite the newer gnomAD (from 241m small variants) frequencies for this SNP (South Asian: 654/30614= 0.02136 with 16 homozygotes; European 91/129152= 0.0007046 with 0 homozygotes). Again, this is evidence that there is no enrichment of this SNP in South Asian PWS.
GNPTG c.802A>C (p.Ile268Leu) is a de novo heterozygous in 1 of 4 pedigree STU63. NAGPA c.131G>C (p.Arg44Pro) does not cosegregate in its pedigree STU34. These results are consistent with the published reports of GNPTAB founder mutation in stuttering South Asians as well as reports that stuttering is polygenic, heterogeneous, and scarcely explained by highly penetrant mutations in the four reported recurrently mutated genes.

This genetic architecture justifies searching whole exomes for other candidate genes.

Scope

Do the research, methods or topics fit within the aims of this, or another journal?
The results are region-specific, underpowered and consistent with uncertain reports of association of these genes with stuttering in some families, but falls short of being confirmatory.

Conceptual advance

What is already known in this area and related fields?
Family specific variants cosegregate with most affected individuals, but allele frequency of founder mutations may be confounders in several populations. Unknown whether haploinsufficiency might explain effects of heterozygous variants. Homozygous variants not found here but conclusions may be confounded by extensive consanguinity in other populations.

1st Peer Review	16-Oct-2020 to 02-Nov-2020

Reviewer #1

The study presents a genetic analysis of 64 unrelated people who stutter, in order to identify possible causal mutations in three previously reported candidate genes for stuttering. The genetic cause of stuttering is highly understudied, including the role of the three candidate genes. This study will therefore be of high interest to researchers studying the genetics of stuttering. I

would also like to applaud the authors for the functional evaluation of the c802A>C variant in GNPTG. However, the pathogenicity of the variants and a likely causative role, although suggested by the authors, cannot be proven by the presented study.

Major comments

1.1 Three variants are classified as "likely pathogenic" by the authors in the majority of the manuscript (c.3598G>A in GNPTAB, c.802A>C in GNPTG and c.131G>C in NAGPA). However, it is not clear what this classification is based on. Actually, the classification by VarSome, that is used to classify the variants according to the Methods section, classifies all variants as (likely) benign or VUS. More importantly, the authors mention data that would classify all three variants as benign:
- The c.131G>C variant does not cosegregate with stuttering in the family. In paragraph 4 of the Results section, the authors write that therefore this variant should not be classified as likely pathogenic.
- The functional studies do not find an effect of the c.802A>C variant in GNPTG
- The frequency of the c.3589G>A variant in GNPTAB in South Asians according to ExAC (MAF=0.02) makes it highly likely to identify this variant once or more often in a cohort of 64 probands

Please use a suitable classification method, for example as described in the ACMG guidelines, that integrates all data gathered in the article, and add a detailed explanation of the utilized classification method in the Methods section. Then use the result of that classification method throughout the manuscript.

1.2 The authors mention multiple times that "the recurrence of the pathogenic variants in lysosomal pathway genes in our study corroborates the causative role for them in stuttering" (in the abstract, discussion and conclusion). However, any group of people will carry several rare variants in the 12 exomes studied. The authors do not provide any evidence that they identify more variants than would be expected by chance, or variants that affect the function of the three proteins more than would be expected. Therefore the authors should:
- Either remove this claim from the manuscript, and instead describe that the study is only a description of the identified variants and that no conclusions can be drawn.
- Or add analyses that may prove a causal role of the identified variants. For example a comparison with variants identified in a cohort of matched controls or mutations generated by simulation using a program such as "dnenrich" (Fromer, et al. Nature, 2014).

1.3 It is unclear how the 12 exons were selected, and why not the full genes were analyzed. Please describe this in the manuscript

Minor comments:

1m1 It would be very helpful to add a general description of the genetic origin of stuttering (i.e. stuttering is a complex disorder, caused by in interplay of many genetic and environmental factors in the majority of cases, as evident from twin studies, and may be monogenic in a subset of cases and families) in the introduction, before describing why it is challenging to study, and the description of the results of published work.
1m2 The introduction is slightly confusing to me because of the information from neuroimaging studies. If the introduction becomes too long because of the addition of a general description of the genetics of stuttering, perhaps the information on neuroimaging results can be removed, or if necessary moved to the discussion.
1m3 The authors write: "There is also growing consensus about the genetic origin of Central Nervous System dysfunctions." (third paragraph of introduction). It is not clear to me what you mean to say here. In addition, the reference (#10) does not describe anything about a genetic origin of CNS dysfunctions
1m4 A role for the four genes in stuttering has indeed initially been identified in Pakistan and Cameroon, as described in the fourth paragraph of the introduction. However, follow-up studies of the same group have studied the role of these genes also in European and American populations.
1m5 The authors describe the results a GWAS study on stuttering in the fifth paragraph of the introduction. However, this study is not published in a peer reviewed journal, only in a thesis, and is not accessible to everyone. I would therefore like to ask the authors to provide more details about the study (including the very small sample size that is not suitable for a GWAS study, and the p-values associated with the mentioned candidate genes), or to remove the mentioning of this GWAS study.
1m6 Please also better describe the mouse models mentioned in the fifth paragraph, for readers not familiar with these papers.
1m7 It is not clear to me what is meant with the following sentence: "Variants observed in NAGPA (n=6) were higher than that of GNPTAB (n=2) and GNPTG (n=4)." (second paragraph of Results section). Please formulate differently.
1m8 Discussion, second paragraph: "We focused on the recurrence of the previously reported mutations in 64 probands". It was not clear to me that your analysis focused only on previously reported mutations. Please make this clear in the Results and the Methods section. If you focused on other variants as well, please remove this sentence.

1m9 Discussion, fourth paragraph: "In order to ..... frequency of 2.2% (4/180*100)." Do I understand correctly that this is a description of an additional analysis you carried out in an additional set of 26 PWS? If so, please add this to the Results section, and add a description of the included patients and used methods to the Methods section.
1m10. If you made Figure 4A based on your own analysis, please describe in the Methods and Results section how Figure 4A was made. Otherwise, please describe in the legend of Figure 4A where the data comes from.
1m11 Discussion paragraph 11 ("If the variation ... mutation in stuttering"). The description of the results of the lysosomal enzyme activity test is slightly confusing to me, because of the use of the word "enzyme" for both the lysosomal enzymes measured in plasma, and GNPTG. It would be very helpful if you could rewrite the paragraph, thereby using "GNPTG" and "lysosomal enzymes" to differentiate between the two types of enzymes involved.
1m12 Discussion paragraph 14: the authors state "there is a preponderance of synonymous and noncoding variants among all the stuttering individuals screened". However, the authors do not present statistical proof for this claim. Therefore please rewrite or remove it to match the results.
1m13 Please describe in the Methods section that family members of the probands were included in the study.

Reviewer #2
Nandhini et al. describe a exploration of genetic variants in PWS, with a focus on specific exons in three genes, previously implicated in stuttering (GNPTAB, GNPTG, NAGPA). Through Sanger sequencing, they identify 12 variants across 64 probands. They classify three of these variants as likely pathogenic, and look at segregation of these variants within the respective proband's families. The most notable finding in the paper is identification of a de novo variant in GNPTG in one proband; however the significance of this variant is uncertain.

I have a number of major comments around the clarity of the descriptions and reporting of results:

2.1 In table 1, are the ExAC frequencies truly 0? Why have ExAC frequencies been shown, and not GnomAD? GnomAD v.2 supersedes ExAC, and contains approx. double the number of South Asian individuals. It would be helpful to included allele counts from ExAC/gnomAD where variants are very rare, and please show very low frequencies in scientific format.

2.2 Figure 2 is difficult to read, and not the most effective way to show these data. I would recommend using an UpSet plot (see https://caleydo.org/tools/upset/) – this will also allow the reader to see combinations of variants.

2.3 "Only three unrelated probands … harbored heterozygous likely pathogenic missense variants" – what criteria were used to classify these as "likely pathogenic"? The ACMG guidelines show these three as VUS or likely benign.

2.4 The authors describe "overall frequencies" and "allele frequencies" of "likely pathogenic missense variants" across all probands. These summaries are not particularly helpful on their own. How many variants would be seen in a control population? How many variants would we expect to see in the 64 probands across these exons?

2.5 "The two missense variants (c.139C>T & c.1394 C>T) in NAGPA, had low conservation scores and were found in high frequency in the ExAC database supporting their benign nature." c.139C>T is seen in 92 South Asian individuals in gnomAD v2, giving an allele frequency of 3.87x10-3. This variant cannot really be said to occur at a high frequency; indeed, it has a similar frequency to c.131G>C in NAGPA, and has a far lower frequency than c.3598G>A in GNPTAB (0.021 in South Asian individuals in gnomAD v.2.1).

2.5 The follow up in the STU 63 Family requires some justification:
- Mucolipidosis III Gamma is a recessive disorder. Given the variant was heterozygous in the proband, what was the rationale for undertaking the lysosomal enzyme study and mucolipidosis screening test? Furthermore, please provide details of what the mucolipidosis screening test consisted of.
- Why was expression of GNTAB and NAGPA examined in this family, given the proband only had a variant of interest in GNPTG?

2.6 In the discussion it states "We hypothesized that in a cohort with severe stuttering as an endophenotype, there may be an increased chance to identify lysine variants in homozygous condition. In order to verify this we tested additionally, 26 severe PWS, but identified again only heterozygous lysine variants in three of them..." – where are the results for this? Why were these individuals not included in the main analyses?

Minor comments:
2m1 Please provide more information about the stuttering severity ratings. What was the severity of individuals with the reported variants?
2m2 State what the colour coding means in the captions to all tables.

2m3 State the genome build used.

1st Editorial Decision	04-Nov-2020

Editorial decision: Return to the authors for major revision with editorial recommendations and reviewer comments
Editor's understanding of the reviews
Reviewer #1 recommends Major Revision
Reviewer #2 recommends Major Revision

**Reviewer comments**	**Editor recommendation**	**Author reply**	**Changes to Manuscript**
1.1 Please use a suitable classification method, for example as described in the ACMG guidelines, that integrates all data gathered in the article, and add a detailed explanation of the utilized classification method in the Methods section. Then use the result of that classification method throughout the manuscript.<br>2.3 Only three unrelated probands … harbored heterozygous likely pathogenic missense variants" – what criteria were used to classify these as "likely pathogenic"? The ACMG guidelines show these three as VUS or likely benign.	ED1 Report all variants performance by ACMG guidelines.		
1.2 Or add analyses that may prove a causal role of the identified variants. For example, a comparison with variants identified in a cohort of matched controls or mutations generated by simulation using a program such as "dnenrich".<br>1.3 It is unclear how the 12 exons were selected, and why not the full genes were analyzed.<br>2.1 GnomAD v.2 supersedes ExAC, and contains approx. double the number of South Asian individuals<br>2.4 How many variants would be seen in a control population? How many variants would we expect to see in the 64 probands across these exons?	ED2 Use current allele frequencies and loss of function statistics from gnomAD South Asians in a statistically valid burden test to generate a null distribution and report the expected variant burden in the context of this distribution and test. Use the full gene sequences or explain why some exons are not tissue-relevant or technically accessible.		

Author's Response to 1st Review	13-Jan-2021

Reply to the Reviewer(s)' Comments by the Authors:
Please note that the reviewer comment is given in **black**, followed by an author answer in blue.

Reviewer: 1
Comments to the Author

The study presents a genetic analysis of 64 unrelated people who stutter, in order to identify possible causal mutations in three previously reported candidate genes for stuttering. The genetic cause of stuttering is highly understudied, including the role of the three candidate genes. This study will therefore be of high interest to researchers studying the genetics of stuttering. I would

also like to applaud the authors for the functional evaluation of the c802A>C variant in GNPTG. However, the pathogenicity of the variants and a likely causative role, although suggested by the authors, cannot be proven by the presented study.

ANS: We thank the reviewer for the appreciation. Yes the pathogenicity of the c802A>C variant can be proven only by functional studies in cell lines or using animal model.

Major comments

1. Three variants are classified as “likely pathogenic” by the authors in the majority of the manuscript (c.3598G>A in GNPTAB, c.802A>C in GNPTG and c.131G>C in NAGPA). However, it is not clear what this classification is based on. Actually, the classification by VarSome, that is used to classify the variants according to the Methods section, classifies all variants as (likely) benign or VUS. More importantly, the authors mention data that would classify all three variants as benign:
- The c.131G>C variant does not cosegregate with stuttering in the family. In paragraph 4 of the Results section, the authors write that therefore this variant should not be classified as likely pathogenic.
- The functional studies do not find an effect of the c.802A>C variant in GNPTG
- The frequency of the c.3589G>A variant in GNPTAB in South Asians according to ExAC (MAF=0.02) makes it highly likely to identify this variant once or more often in a cohort of 64 probands

ANS: We used Varsome that classifies variants according to ACMG guidelines.

Please use a suitable classification method, for example as described in the ACMG guidelines, that integrates all data gathered in the article, and add a detailed explanation of the utilized classification method in the Methods section.

ANS: The classification is now detailed in the methods section and is pasted below. The edited/added information is given. Page no:15
Variants identified were predicted using VarSome (https://varsome.com/; tools include various predictors like DANN, Mutation taster, Likelihood Ratio Test - LRT, Mutation assessor, SIFT, Provean etc) and Polyphen tool, to deduce the pathogenicity. It was classified according to ACMG guidelines along with the ACMG attributes. The guidelines describe the process of classifying variants into five categories as “pathogenic,” “likely pathogenic,” “uncertain significance,” “likely benign,” and “benign”, based on the evidences from computational data, population data, functional data, segregation data.

Then use the result of that classification method throughout the manuscript.

ANS: Yes we have now used the findings based on the same classification method throughout the manuscript. Page No:5
This paragraph is now changed as shown below.

The three missense variants in the putative genes, with high conservation scores were observed in three unrelated probands resulting in an allele frequency of 2.3% (3/128*100).

2. The authors mention multiple times that “the recurrence of the pathogenic variants in lysosomal pathway genes in our study corroborates the causative role for them in stuttering” (in the abstract, discussion and conclusion). However, any group of people will carry several rare variants in the 12 exons studied. The authors do not provide any evidence that they identify more variants than would be expected by chance, or variants that affect the function of the three proteins more than would be expected. Therefore the authors should:
- Either remove this claim from the manuscript, and instead describe that the study is only a description of the identified variants and that no conclusions can be drawn.

ANS: Yes this 'claim' is now removed. Instead the passage reads as follows.
Although the study has identified some of the reported variants, that may have crucial role, no conclusions could be drawn as to how the speech dysfluency occurs in heterozygous condition.
(Page no:2)

Although our the study has identified some of the reported variants, the perplexing question as to how the speech dysfluency occurs even in its heterozygous condition still remains. (Page no:13)

Conclusion: Recurrence of mutations in the three genes among our south Indian stuttering cohort corroborates the causative of these genes to stuttering.

This study report the recurrence of some of the previously reported variants yet the causative role in stuttering disorder as opposed to the recessive nature of these genes remains elusive. While not delimiting our observation from the results of this study, we emphasize the need for similar studies to evaluate on the heterozygous nature of variants in genes of lysosomal pathway. . (Page no:13)

- Or add analyses that may proof a causal role of the identified variants. For example a comparison with variants identified in a cohort of matched controls or mutations generated by simulation using a program such as “dnenrich” (Fromer, et al. Nature, 2014).

ANS: We tried using the suggested dnenrich program. However it was not suitable for this data because: 'dnenrich' is a statistical package for calculating gene set enrichment especially for exome data. This package is not suitable for this study as the variants status is described using Sanger sequencing which is by itself a confirmatory test.

3. It is unclear how the 12 exons were selected, and why not the full genes were analyzed. Please describe this in the manuscript

ANS: Yes. It is now addressed in the manuscript as follows: (shown in red). Page No: 14
The 12 specific exons spanning across the three genes viz., GNPTAB, GNPTG and NAGPA implicated in stuttering were screened (Figure 1). Our analysis was focused to investigate on the known and any novel variants that occurred in these twelve exons studied. Primer sequences were adapted from Kang et al., (2010), after improvising (NCBI's Primer-BLAST) the sequence coverage of exon 10 of NAGPA gene. As a cost effective approach we selected these 12 exons to observe the recurrence of mutation in our ethnic population that is unexplored till this date.

Minor comments:

1. It would be very helpful to add a general description of the genetic origin of stuttering (i.e. stuttering is a complex disorder, caused by in interplay of many genetic and environmental factors in the majority of cases, as evident from twin studies, and may be monogenic in a subset of cases and families) in the introduction, before describing why it is challenging to study, and the description of the results of published work.

ANS: Yes we fully agree to this and this general description on the genetic origin of stuttering has now been incorporated. In the introduction section we have now added this as follows: (Page No 2)
" Stuttering is a complex disorder caused by interplay of genetic and environmental factors in a majority of cases, as evident from twin studies with varied heritability(5). The Genetic epidemiological studies provided inconsistent evidence on the modes of inheritance, yet revealed familial clustering of the disorder and may be monogenic in a subset of cases and families, rendering stuttering as a complex genetic trait (7–9)".

2. The introduction is slightly confusing to me because of the information from neuroimaging studies. If the introduction becomes too long because of the addition of a general description of the genetics of stuttering, perhaps the information on neuroimaging results can be removed, or if necessary moved to the discussion.

ANS: The information on neuroimaging results in the introduction is now removed so that the introduction does not become too long.

3. The authors write: “There is also growing consensus about the genetic origin of Central Nervous System dysfunctions.” (third paragraph of introduction). It is not clear to me what you mean to say here. In addition, the reference (#10) does not describe anything about a genetic origin of CNS dysfunctions

ANS: Yes we agree and so this passage is also now removed in the introduction.

4. A role for the four genes in stuttering has indeed initially been identified in Pakistan and Cameroon, as described in the fourth paragraph of the introduction. However, follow-up studies of the same group have studied the role of these genes also in European and American populations.

ANS: Yes now we have added a line about the follow up studies in the European and American populations along with reference as follows: (Page No 3)

Although linkage studies are spread across Hutterite, European and American population, the four genes, GNPTAB, GNPTG, NAGPA and AP4E1, identified are restricted to two regions [Pakistan19; Cameroon20] with distinct ethnicities. However, follow-up studies by the same group have studied the role of these genes in European and American populations 20)

5. The authors describe the results a GWAS study on stuttering in the fifth paragraph of the introduction. However, this study is not published in a peer reviewed journal, only in a thesis, and is not accessible to everyone. I would therefore like to ask the authors to provide more details about the study (including the very small sample size that is not suitable for a GWAS study, and the p-values associated with the mentioned candidate genes), or to remove the mentioning of this GWAS study.

ANS: We have retained the GWAS study but provided more details about the study including the sample size and p value range associated with the mentioned candidate genes (shown in red). It now reads as follows: (Page No.4)
A GWAS study of stuttering, investigated 84 subjects with age ranging from 13-70 years of northern European ancestry, to identify candidate genes that influence the risk of developing stuttering. With limited statistical power, the study suggested ten candidate genes (FADS2, PLXNA4, CTNNA3, ARNT2, EYA2, PCSK5, SLC24A3, FMN1, ADARB2 and non-coding RNA RNU6-259P) with a p-value less than 10-4 and greater than 10-8, involved in neural pathways23.

6. Please also better describe the mouse models mentioned in the fifth paragraph, for readers not familiar with these papers.

ANS: The recent paper on mouse model is described in depth in the introduction section as suggested and is shown (Page No:3)
Since none of the genes identified have an obvious connection to speech, Drayna's team performed mice model studies that linked the implicated genes to brain's activity. Firstly the GNPTAB gene mutation (Glu1200Lys) was engineered in mice to observe any change in mice vocalization. Mice vocalization is in ultrasonic range and hence recording the ultrasonic calls of pups showed patterns of gaps and pauses that are similar to human stuttering. On further probing to spot the clear defect, they found fewer cells of astrocytes in corpus callosum that slows down the communication between hemispheres by a tiny bit that can be noticed only in speech. 24,25

7. It is not clear to me what is meant with the following sentence: "Variants observed in NAGPA (n=6) were higher than that of GNPTAB (n=2) and GNPTG (n=4)." (second paragraph of Results section). Please formulate differently.

ANS: The above sentence is reframed and is also shifted from the second paragraph to the fourth paragraph of the results section to have better continuity and reads as follows (Page No:5)

The number of variants observed in NAGPA (n=6) were more than that observed in GNPTAB (n=2) and GNPTG (n=4)

8. Discussion, second paragraph: "We focused on the recurrence of the previously reported mutations in 64 probands". It was not clear to me that your analysis focused only on previously reported mutations. Please make this clear in the Results and the Methods section. If you focused on other variants as well, please remove this sentence.

ANS: Thank you for this valuable remark. We have now corrected this and the sentence reads as follows: (Page No.9)

To the best of our knowledge this is the first study from India to investigate three functionally related genes *viz., GNPTAB*, *GNPTG* and *NAGPA* implicated in stuttering. We screened 12 specific exons where the variants have been previously reported. Our analysis focused not only on the previously reported variants but also on any other variants that occurred in these twelve exons studied.

9. Discussion, fourth paragraph: "In order to ..... frequency of 2.2% (4/180*100)." Do I understand correctly that this is a description of an additional analysis you carried out in an additional set of 26 PWS? If so, please add this to the Results section, and add a description of the included patients and used methods to the Methods section.

ANS: Yes. It is the description of an additional set of 26 persons with severe stuttering. The analysis is now been explained in the results section and the description of the included patients is also given in the methods section as follows:

Results: The segregating lysine variant (p.Glu1200Lys) in *GNPTAB* gene was screened in an additional subset of 26 persons with severe stuttering collected during the annual meeting of a Self help group for PWS. Three of them were found to carry the lysine variant increasing this allele frequency from 0.8% (1/128*100) to 2% (4/180*100). Page No:7

Methodology: An additional subset of 26 severe stuttering adults were recruited from a stuttering self help group annual conference whose extended family members' study was not possible. This subset was utilized interim to answer a specific question during the progress of the work. Page No 14

10. If you made Figure 4A based on your own analysis, please describe in the Methods and Results section how Figure 4A was made. Otherwise, please describe in the legend of Figure 4A where the data comes from.

ANS: Figure A4 was constructed based on our own analysis. This clarification is now given in methods and results section and also hinted in the legend of A4. We have also added the bioinformatic analysis for another variant in GNPTG gene. The figures are now labelled as figure A4a and A4b that is placed in the appendix. The revised content reads as follows:

Methodology: Effects of E1200K and I268L amino acid change in GNPTAB and GNPTG protein respectively:
Both the native protein and mutated protein of GNPTAB and GNPTG was subjected to pair wise alignment using the Geneious Pro version 6.1.2. The pair wise alignment was carried out by MAFFT alignment. Default parameters were set to assess and predict the effect of SNP identified in this study. Page No 15
Results: Bioinformatic analysis of the alignment of secondary structure of mutated GNPTAB protein (Glu1200Lys) with the native protein identified loss of helix and addition of turn near the mutated region (figure A4a). But for GNPTG protein the mutation does not affect the secondary structure (figure A4b). Page No 8

11. Discussion paragraph 11 ("If the variation ... mutation in stuttering"). The description of the results of the lysosomal enzyme activity test is slightly confusing to me, because of the use of the word "enzyme" for both the lysosomal enzymes measured in plasma, and GNPTG. It would be very helpful if you could rewrite the paragraph, thereby using "GNPTG" and "lysosomal enzymes" to differentiate between the two types of enzymes involved.

ANS: Yes the paragraph starting with "If the variation ... mutation in stuttering" is now rewritten to address the confusion caused by the use of the word 'enzyme'. We have used the word lysosomal and GNPTG enzymes as follows: Page No 12

If the variant affects the targeting function, the lysosomal enzymes will not be targeted to lysosomes but will be secreted in plasma. Thus the enzyme deficiency can be demonstrated by elevated lysosomal enzyme activity in plasma[31]. Nevertheless, in our study the activity of ~~lysosomal~~ GNPTG enzymes were not elevated in plasma, indicating that ~~the enzyme~~ it might be successfully targeted to lysosomes. Also the proband with stuttering did not have any symptoms of mucolipidosis and tested negative. We propose that, since the ~~variation~~ variant observed is in heterozygous condition, either the normal copy is sufficient or this variation does not affect the function of the enzyme. Similarly, there was no fold change in the mRNA level of the three genes between the affected (proband) and unaffected members (father, mother and sister) of the family. Hence it was difficult to conclusively demonstrate the pathogenicity of this *de novo* mutation in stuttering.

12. Discussion paragraph 14: the authors state "there is a preponderance of synonymous and noncoding variants among all the stuttering individuals screened". However, the authors do not present statistical proof for this claim. Therefore please rewrite or remove it to match the results.

ANS: This is another valuable comment. We have removed the above sentence besides adding the statistical proof for the variants associated with stuttering. We have used the odds ratio test and incorporated in table 6 and also in the text that is now reframed. PageNo:12

One synonymous (c.1932A>G) and one non coding variant(-4 C>T) was significantly associated with stuttering as demonstrated by odds ratio (table 6). However the two other variants (c.813G>A and c.1174+53C>A) observed in our affected cohort was not found in south Asian gnomAD data. So the role of the commonly occurring synonymous (five) and non coding variants (two) that was observed in our study among all the stuttering individuals cannot be ignored. Some of them are also seen to co-occur with the conserved missense variants.

Table 6: Strength of association of identified variants with stuttering Page No 23

Gene	Nucleotide change	Variants in PWS (No of alleles)	Variants in gnomAD controls (No of alleles)	PWS without variant (No of alleles)	gnomAD controls without mutation (No of alleles)	Crude Odds ratio	95% CI	Significance

Non synonymous variants								
GNPTAB	c.3598G>A	1	654	127	29960	0.3607	0.0503 to 2.5845	P = 0.3102
GNPTG	c.802A>C	1	1	127	30613	241.0472	14.9942 to 3875.0939	P = 0.0001
NAGPA	c.131G>C	1	84	127	29376	2.7537	0.3805 to 19.9305	P = 0.3158
NAGPA	c.139C>T	1	0	127	23756	-	-	-
NAGPA	c.1394 C>T	22	5358	106	25242	0.9778	0.6172 to 1.5491	P = 0.9237
Synonymous variants								
GNPTAB	c.1932A>G	96	19088	32	11522	1.8109	1.2130 to 2.7035	P = 0.0037
GNPTG	c.702T>C	1	109	127	30505	2.2036	0.3053 to 15.9073	P = 0.4334
GNPTG	c.813G>A	18	0	110	30614	-	-	-
NAGPA	c.333 A>G	101	22109	27	4781	0.8089	0.5284 to 1.2383	P = 0.3290
NAGPA	c. 1485C>T	106	25034	22	5582	1.0743	0.6781 to 1.7020	P = 0.7600
Non coding variants								
GNPTG	-4 C>T	1	1	127	3699	29.1260	1.8115 to 468.3085	P = 0.0173
NAGPA	c.1174+53C>A	70	0	58	0	-	-	-

13. Please describe in the Methods section that family members of the probands were included in the study.

ANS: Yes this sentence has now been included and reads as: (shown in red) Page No 14

Eight milliliters of blood was collected from probands by venipuncture into labelled EDTA coated vacutainers (Beckon and Dickinson Co., USA). Genomic DNA was isolated using Phenol-Chloroform extraction method[35]. The family members of the probands were included in the study.

Reviewer: 2

Comments to the Author

Nandhini et al. describe a exploration of genetic variants in PWS, with a focus on specific exons in three genes, previously implicated in stuttering (GNPTAB, GNPTG, NAGPA). Through Sanger sequencing, they identify 12 variants across 64 probands. They classify three of these variants as likely pathogenic, and look at segregation of these variants within the respective proband's families. The most notable finding in the paper is identification of a de novo variant in GNPTG in one proband; however the significance of this variant is uncertain.

ANS: Yes the pathogenencity of the c802A>C variant that occurred *de novo* can be proven only when functional studies in cell lines or in animal model are conducted.

I have a number of major comments around the clarity of the descriptions and reporting of results:

• In table 1, are the ExAC frequencies truly 0? Why have ExAC frequencies been shown, and not GnomAD? GnomAD v.2 supersedes ExAC, and contains approx. double the number of South Asian individuals. It would be helpful to included allele counts from ExAC/gnomAD where variants are very rare, and please show very low frequencies in scientific format.

ANS: Yes the frequency was truly 0 and was also verified onceagain. The ExAC frequencies are now replaced with more updated gnomAD frequencies in the table 1 as suggested (pasted below and shown in red). We have newly added a table (table 6) that shows the allele counts for all the variants identified in our study.

Table 1: Allele frequencies of the 12 variants observed in *GNPTAB, GNPTG* and *NAGPA* genes among the 64 probands with stuttering and their comparison with gnomAD database (Page No:19)

S.No	GENE	Nucleotide change	Amino acid change	Exon	dbSNP ID	Hom/Het (n=64 probands)	Allele frequency (n=128 alleles)	Stuttering studies	Allele frequency in South Asian gnomAD	Allele frequency in total gnomAD
**Missense variants**										
1	*GNPTAB*	**c.3598G>A**	Glu1200Lys	Exon 19	rs137853825	- /1	0.008	(8/1013cases) = 0.00789	0.0214	**0.0028**
2	*GNPTG*	**c.802A>C**	Ile268Leu	Exon 10	rs759796840	- /1	0.008		0.00003266	**0.000003979**
3	*NAGPA*	**c.131G>C**	Arg44pro	Exon 2	rs374266430	- /1	0.008	(1/1013) = 0.000099	0.002851	**0.0003922**
4	*NAGPA*	c.139C>T	Leu47Phe	Exon 2	rs371054576	- /1	0.008		0.000	**0.00001123**
5	*NAGPA*	c.1394 C>T	Thr465Ile	Exon 10	rs7188856	- /22	0.172		0.1751	**0.2990**
**Synonymous variants**										
6	*GNPTAB*	**c.1932A>G**	Thr644Thr	Exon13	rs10778148	42/12	0.75	120/1708 alleles	0.6236	**0.5900**
7	*GNPTG*	**c.702T>C**	Pro234Pro	Exon 9	rs532275192	- /1	0.008		0.003560	**0.0004377**
8	*GNPTG*	**c.813G>A**	Thr271Thr	Exon 10	rs377647926	- /18	0.14		0.000	**0.00001771**
9	*NAGPA*	**c.333 A>G**	Gly111Gly	Exon 2	rs2972272	41/19	0.789	229/1708	0.8222	**0.6981**
10	*NAGPA*	**c. 1485C>T**	Asn495Asn	Exon 10	rs887854	42/22	0.828		0.8177	**0.6967**
**Non coding variants**										
11	*GNPTG*	**-4 C>T**	-	5'UTR	rs554707396	- /1	0.008		0.0002703	**0.00006478**
12	*NAGPA*	**c.1174+53C>A**	-	intron 7	rs2937112	22/26	0.547		0.000	**0.2792**

• Figure 2 is difficult to read, and not the most effective way to show these data. I would recommend using an UpSet plot (see https://caleydo.org/tools/upset/) – this will also allow the reader to see combinations of variants.

ANS: Thank you for this valuable comment. We have now used the UpSet plot that would allow the reader to see combination of variants. Figure2 is now replaced by the UpSet plot and explained in the freshly added text under the results section that is shown below: (Page No 5

The identified variants and their interactions were analyzed in an upset plot using R program (Figure 2). It visualizes intersections of sets as a matrix in which the row represents variants and the columns represent the number of probands having that particular combination of variants. Thus each identified variant represents a set and each proband represents an element that is contained in one or more sets. We observed that all the probands had Asn495Asn synonymous variant in the *NAGPA* gene in common, along with atleast two or more other synonymous variants. Variants like p.Glu1200Lys in *GNPTAB*, 5' UTR, p.Pro234Pro and p.Ile268Leu in *GNPTG* and p.Arg44Pro and p.Leu47Phe in *NAGPA* gene, were found in isolated cases.

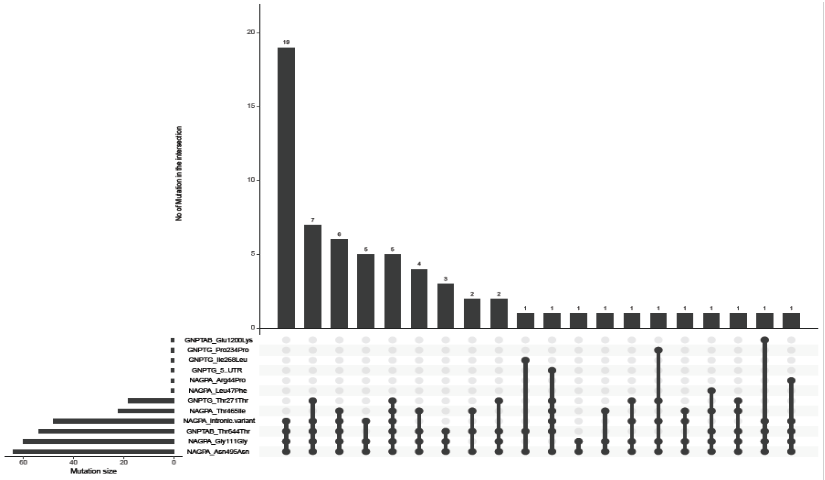

Figure 2: An Upset plot of identified variants in this study across three genes implicated in stuttering to show combination of variants among probands. For each variant that is a part of intersection, a black filled circle is shown in the matrix and for the variant that is not part of intersection, a light gray circle is placed. The number of probands bearing the set of variants (column-based relationships) is emphasized by vertical black line connecting the top most and bottom most black circle. The bar chart on top and left of the matrix gives the size of intersections and sets respectively.

• "Only three unrelated probands … harbored heterozygous likely pathogenic missense variants" – what criteria were used to classify these as "likely pathogenic"? The ACMG guidelines show these three as VUS or likely benign.

ANS: This an important question asked by both the reviewers. Yes we have now used the findings based on the same classification method throughout the manuscript in an uniform manner.

• The authors describe "overall frequencies" and "allele frequencies" of "likely pathogenic missense variants" across all probands. These summaries are not particularly helpful on their own. How many variants would be seen in a control population? How many variants would we expect to see in the 64 probands across these exons?

ANS: Yes we agree the summaries are not particularly helpful on their own. We have removed the term overall frequencies that occurs in three places in the manuscript. In addition we have now stated the details about the allele frequencies observed in the gnomAD database which has been considered as controls and this sentence is now added in results section (pasted below and shown in red). The variants observed in 64 probands across these exons are also given in table 1. Page No 7

In the control (gnomAD database) the allele frequency of this variant was 2.1%. Also this variant is not significantly associated with stuttering (table 6).

• "The two missense variants (c.139C>T & c.1394 C>T) in NAGPA, had low conservation scores and were found in high frequency in the ExAC database supporting their benign nature." c.139C>T is seen in 92 South Asian individuals in gnomAD v2,

giving an allele frequency of 3.87x10-3. This variant cannot really be said to occur at a high frequency; indeed, it has a similar frequency to c.131G>C in NAGPA, and has a far lower frequency than c.3598G>A in GNPTAB (0.021 in South Asian individuals in gnomAD v.2.1).

ANS: Though the *NAGPA* variant c.139C>T occurs at a frequency lower than *GNPTAB* variant c.3598G>A, its prediction is not pathogenic and is also not conserved across species. The text is now revised and reads as follows: Page No 5

Two additional missense variants (p.Leu47Phe & p.Thr465Ile) observed in *NAGPA,* showed a low conservation score and the variants had benign predictions. Also the gnomAD exome allele frequency is greater than the required threshold confirming their benign nature. Hence, segregation analysis and genotype-phenotype correlations were performed only for the three missense variants with high conservation score.

- The follow up in the STU 63 Family requires some justification:

- Mucolipidosis III Gamma is a recessive disorder. Given the variant was heterozygous in the proband, what was the rationale for undertaking the lysosomal enzyme study and mucolipidosis screening test?

ANS: The rationale for mucolipidosis screening test is now added in the results section and is shown below. Page No 8

To study the impact of a *de novo* heterozygous variant (c.802A>C/+) in *GNPTG* gene identified in one family (STU 63), mRNA expression profile and lysosomal enzyme study was performed along with mucolipidosis screening test. If one looks at the pedigree, the pattern of inheritance in this family, signifies a dominant mode hinting onto investigating the importance of heterozygosity and its relevance in dysfluency. But in the case of mucolipidosis III it follows a recessive mode of inheritance. In the light of this, the rationale for evaluating the lysosomal enzyme assay was set for determining the differences in the plasma level though not in terms of severe morbidity.

Furthermore, please provide details of what the mucolipidosis screening test consisted of.

ANS: The details of mucolipidosis screening is given now in the methods section and reads as below. Page No: 16

All members in the family were also evaluated for mucolipidosis phenotype using a rapid calorimetric screening method. It is a simple chemical test where the synthetic substrate pNCS (p-nitrocatechol sulphate) gets hydrolysed in presence of Arylsulfatase-A (ASA) when excessively present in the plasma and catalyzes to form excess pNC. It gives dark brown colour in alkaline solution which is visible to the naked eye

- Why was expression of GNTAB and NAGPA examined in this family, given the proband only had a variant of interest in GNPTG?

ANS: The rationale in choosing *GNPTAB* and *NAGPA* is now explained and pasted below. Page No 8

We quantified all the three genes to examine whether the defect in *GNPTG* gene affect expression of other components, *GNPTAB* and *NAGPA*, invovled in mannose 6 phosphate formation[26]. Further *GNPTG* and *GNPTAB* genes encode different subunits of the same enzyme and there is a possible feedback regulation mechanism between them[27].

- In the discussion it states “We hypothesized that in a cohort with severe stuttering as an endophenotype, there may be an increased chance to identify lysine variants in homozygous condition. In order to verify this we tested additionally, 26 severe PWS, but identified again only heterozygous lysine variants in three of them...” – where are the results for this? Why were these individuals not included in the main analyses?

ANS: Yes. It is the description of an additional set of 26 persons with severe stuttering. The analysis is now been explained in the results section and the description of the included patients is also given in the methods section as follows: (shown in red)

Results: The segregating lysine variant (Glu1200Lys) in *GNPTAB* gene was screened in an additional subset of 26 persons with severe stuttering collected during the annual meeting of a Self help group for PWS. Three of them were found to carry the lysine variant increasing this allele frequency from 0.8% (1/128*100) to 2% (4/180*100). (Page No:8)
Methodology: An additional subset of 26 severe stuttering adults were recruited from a stuttering self help group annual conference whose extended family members' study was not possible. This subset was utilized interim to answer a specific question during the progress of the work. (Page No:14)

Minor comments:

- Please provide more information about the stuttering severity ratings.

ANS: Stuttering severity was rated using SSI-3. We have now provided more information about the parameters used to rate the severity in the methodology section and is shown below (Page No:14)

It measures stuttering severity using three parameters: (a) frequency of stuttering, expressed as a percentage of words stuttered, (b) duration which is the average of the three longest stuttering moments, and (c) recognizable physical concomitants, culminating in a single total overall score.

What was the severity of individuals with the reported variants?

ANS: This is an important question and actually gives scope to present the genotype-phenotype correlations. For want of space in the first draft of the manuscript we refrained from presenting the details about the severity and segregation analysis for the identified variants. We have now added this in detail and it reads as follows: (Page No: 6)

Segregation analysis and genotype-phenotype correlations:

Family STU 29

The 16-year-old proband with stuttering was ascertained from a government boys higher secondary school in Salem, Tamil Nadu. He was born to non consanguineous parents and had no complications during his birth. His age of onset of stuttering was reported to be 3 years and he was right-handed. Severity assessment rated him as severe with excess of prolongations, blocks and difficulties in initial syllables. Secondary behaviours include eye blinking, stiffness of the body, tension in neck and avoidance of eye contact. There was a situational increase in the stuttering such as in a classroom, while speaking with teachers, superiors or opposite sex and when excited/afraid. Both the proband's father and his elder brother had stuttering that could be rated as moderate.

Mutational analysis in the proband identified a heterozygous conserved missense variant p.Glu1200Lys in exon 19 of *GNPTAB* gene. Both his affected father and affected brother also had the same variant in the heterozygous condition. The proband's unaffected mother and his three unaffected siblings did not carry this variant. The cosegregation of the mutant allele with the affected status suggests a dominant pattern of inheritance (Figure 3). The impact of this mutation in the relative quantification of the mRNA using real-time PCR could not be done since this family relocated and was not traceable.

Family STU 63

This 24-year-old male proband was referred to our unit for genetic counseling. His mother informed that his speech was normal until a sudden onset at the age of nine. Severity assessment rated him as mild with repetitions, blocks and had eye closure during speech. The proband was born to non-consanguineous parents without any complications during birth. He was right-handed with good academic performance.

Mutation analysis of the proband identified a missense heterozygous variant p.Ile268Leu in exon 10 of *GNPTG* gene. On extending the analysis to the nuclear family, his unaffected father, mother and sister did not show this variation. Since this variant is not present in either of the parent but only observed in the proband, it is termed *de novo* (figure 4). Since the *de novo* mutation occurred in a heterozygous condition in the affected, we assume that it is dominant.

Family STU 34

The 15-year-old proband with stuttering was ascertained from a National high school in Salem, Tamil Nadu. He was born to non consanguineous parents and had no complications during his birth. The age at onset was unknown and reported as sudden. He is right-handed with good academic performance. Severity assessment rated him as severe with prolongations, blocks, irregular breathing and with difficulties in initial syllables. Secondary behaviours were mild. There was a situational increase in the stuttering such as in a classroom, while speaking with teachers, etc.

Mutation analysis identified a heterozygous variant p.Arg44Pro in exon 2 of *NAGPA* gene. When the analysis was extended to the family members even the unaffected father and his two brothers harbored the variant. Since the variant is present in both affected and unaffected the pathogenicity is inconclusive (Figure 5).

Table 3 shows a comprehensive variant profile for the three putative stuttering genes for all the three proband presented above. The three putative genes so far reported for stuttering are functionally related and belong to the same lysosomal targeting pathway. On examining the variant profiles of these three probands (STU 29, STU 63 and STU 34) there was a co-occurrence of synonymous and non-coding variants in all of them.

Thus, the segregation analysis revealed that only one variant, p.Glu1200Lys in *GNPTAB* gene, co-segregated with the affected individual (table 4) while p.Ile268Leu in *GNPTG* gene was found to be a *de novo*, thus reducing the allele frequency to 1.6% (2/128).

- State what the colour coding means in the captions to all tables.

ANS: The colour coding is now removed as it does not add much

- State the genome build used. (Page No:15)

ANS: The genome build used is GRCh37/hg19 build which is now mentioned in the methods section

2nd Peer Review	24-Jan-2021 to 09-Feb-2021

Reviewer #1

I would like to thank the authors for working with my comments to adjust the manuscript. However, I have a few comments on the new additions, especially on the newly added comparison of allele frequencies.

Major comments
1.1 A new analysis was included in the manuscript to compare allele frequencies of the identified variants between the study cohort and the gnomAD database (Table 6).
- No details on the statistical analysis are provided in the material and methods section. Please add them.
- The huge difference in sample size between the study cohort and gnomAD make it difficult to properly compare the allele frequencies between the samples especially for rare variants. This is reflected in the significant results for several of the variants compared, for example GNPTG c.802A>C with frequencies 1/127 vs 1/30613 gives a highly significant result. Please use a statistical test that can deal with this difference in sample size, or correct by for example drawing random subsets of gnomAD with a similar sample size. You will see that 1/127 vs 0/127 does not give a significant difference.
- It is very difficult to do such a comparison with a public control dataset when looking at rare variants or variants with different allele frequencies between populations. Please describe properly what has been done to assure that the control cohort is comparable regarding ancestry before performing this analysis.

1.2 You make use of a very good and reliable method to classify your variants as pathogenic or benign, or anything in between. Yet you do not yet integrate its results properly into your manuscript:
- In the results section on Family STU 63, you write “Since the de novo mutation occurred in a heterozygous condition in the affected, we assume that it is dominant.” However, the variant is predicted to be benign. Please adapt accordingly.
- Results section, section about Family STU 34 (page 23, paragraph 3): You write that the pathogenicity of the variant p.Arg44Pro in NAGPA is inconclusive. However, according to table 2, this variant is “likely benign”. Please adapt the text accordingly.
- In the discussion section, page 26, paragraph 2, you discuss c.3598G>A in GNPTAB. You write that the high allele frequency of this variant in Gnomad “questions its pathogenicity.” In paragraph 3 on the same page, you write again about “conflicting evidence for pathogenicity”. However, in Table 2, this variant is classified as “benign”. Please adapt the text accordingly.
- In the discussion section, you discuss the c.131G>C variant in NAGPA. You mention “Since its frequency was also low in gnomAD database the role of this variant remains inconclusive.”. However, VarSome scored this variant as “likely benign”. Please adapt the text accordingly.
- The titles of Table 3 and Table 4 and a remark in Table 4 describe three variants as pathogenic variants. However, these variants are classified as benign or likely benign by VarSome. Please adapt accordingly.

Minor comments
1m1 At several locations (abstract paragraph 2; results section about Family STU 34) you write "p.Ile268Leu in GNPTG gene was found to be a de novo, thus reducing the allele frequency to 1.6% (2/128)." I don't understand of what allele the frequency is lowered, and why.

1m2 Page 19, paragraph 3: "The combined contribution of these genes were estimated to be 20% and all of them point to intracellular trafficking deficits(20)." Later articles have lowered this estimation to 12%. See for example Frigerio-Domingues C, Drayna D. Genetic contributions to stuttering: the current evidence. Mol Genet Genomic Med. 2017 Feb 19;5(2):95-102.

1m3 Page 19, paragraph 4: "However, family based linkage studies till date did not report any significant signal at this location." Please explain in what locus. For example, the GNPTAB locus showed significant linkage with stuttering (Kang C, Riazuddin S, Mundorff J, Krasnewich D, Friedman P, Mullikin JC, Drayna D. Mutations in the lysosomal enzyme-targeting pathway and persistent stuttering. N Engl J Med. 2010 Feb 25;362(8):677-85.)

1m4 Page 20, paragraph 2: you describe a GWAS study on stuttering in 84 individuals. Please comment that p>10e-8 is not significant after correction for multiple testing.

1m5 The newly added text on segregation analysis and genotype-phenotype correlations is very long. Especially when describing three variants that are predicted to be benign or likely benign. Perhaps it can be summarized or partially moved to the supplement.

1m6 You write "In fact, dominance and recessiveness are not essentially allelic properties but measured in relation to the effects of other alleles at the same locus." In the discussion section (page 26, last sentence). Please provide a reference for this statement, and perhaps more information, as it is a rather difficult concept.

1m7 You write in the conclusion that your mutation screening resulted in an allele frequency of 1.6%. Please detail of what allele this is the frequency. In case you mean "likely causal variants" or similar, please consider your VarSome results in Table 2.

1m8 In Table 1, please adjust the allele frequency of rs2937112 in the South Asian gnomAD: there is no data in this subset for this variant, so the allele frequency is not 0 but unknown.

Reviewer #2

The authors have made several updates to the paper, in response to the reviewers' comments. There are still a number of outstanding issues, detailed below. The most interesting finding in the paper remains the identification of the de novo variant in one proband, and the subsequent follow-up. The relevance of the other variants is unclear, and without suitable controls, summaries of the identified variants are very difficult to interpret.

2.1 Please check again the gnomAD frequencies in table 1 and consider adding frequencies from 1000Genomes reference population too.
For example, Variant 4 (c.139C>T in NAGPA) should have an allele count of 92. This site is multiallelic, and the count for a different allele is given:
https://gnomad.broadinstitute.org/variant/16-5083677-G-A?dataset=gnomad_r2_1

Variant 12 (c.1174+53C>A in NAGPA): The gnomAD frequency in South Asians, does indeed appear to be 0; so, to see so many probands with this variant seems strange. Looking at dbSNP, in the 1000Genomes population, this SNP has an allele frequency of 0.57 (A) in the South Asian population. (I am unsure why the frequency in gnomAD was 0.)
https://www.ncbi.nlm.nih.gov/snp/rs2937112#frequency_tab

2.2 I have concerns about the addition of table 6 and what this adds. Firstly, there is no description of the statistical methods used – how were the p-values calculated? Secondly, there are several issues with using summary data from a database as "controls" – see https://doi.org/10.1101/115964 for discussion. This table was seemingly added in response to the comment by both reviewers, that the authors do not provide evidence that more variants would be seen in the probands, than would be expected by chance. I do not think table 6 sufficiently provides this evidence, and it would be better if the authors remove this

from the manuscript.

2.3 The additional detail about the three probands is interesting; The rationale for following up these three probands, and not the other 2 probands with NAGPA variants, remains somewhat weak. It is (vaguely) stated that follow-up was based on conservation score and frequency. The results state “Also the gnomAD exome allele frequency is greater than the required threshold confirming their benign nature.”, but “the required threshold” is not stated anywhere. In the discussion it states “Three of the conserved nonsynonymous variants with less than 1% MAF for segregation analysis that are discussed individually” – so perhaps the threshold is 1%? However, c.3598G>A in GNTAB has a MAF>2% in individuals of South Asian ancestry in gnomAD. Please clarify further criteria for follow-up (I assume this particular variant was selected for follow-up, given that specific variant was previously reported?).

Minor:
Introduction (page 19 in proof) :
2m1“The Genetic epidemiological studies provided inconsistent evidence on the modes of inheritance, yet revealed familial clustering of the disorder and may be monogenic in a subset of cases and families, rendering stuttering as a complex genetic trait” – this sentence seems a little contradictory of itself?
And
2m2 “However, family based linkage studies till date did not report any significant signal at this location.” – I’m unclear what this is refering to?

Results (page 21):
2m3 “The identified variants and their interactions” – this sounds like the authors are suggesting interactions (biological or statistical) – this should be re-worded.

2m4 Legend for table 3 is “Variant profile for the three putative genes for stuttering in probands with pathogenic mutation” – remove mention of “pathogenic mutation”.

2nd Editorial Decision	10-Feb-2021

Editorial decision: Major Revision. Although there has been a revision in good faith, the issues raised at first round of review remain. A standard classification system for variant interpretation has not been systematically implemented throughout the paper. Secondly, although public allele frequencies have been used as controls, the statistical framework and procedures for dealing with inconsistencies in sample size and allele frequency in different populations have not been described in detail. I have made some broad suggestions where there are concordant recommendations from the reviewers, but I do think it is essential to address all of the reviewers’ comments in detail.

Editor’s understanding of the reviews

Reviewer #1 recommends Major Revision, in particular, to detail the statistical procedure to establish the significance of the allele frequency comparisons.

Reviewer #2 recommends caution since there are still no suitable controls for some variants, and the allele frequencies in control populations need to be carefully documented and considered.

Reviewer comments	Editor recommendation	Author reply	Changes to Manuscript
1.1 No details on the statistical analysis are provided in the material and methods section… Please use a statistical test that can deal with this difference in sample size, or correct by for example drawing random subsets of gnomAD with a similar sample size<br>2.2 there is no description of the statistical methods used ... there are several issues	ED1 Use a statistical method that can deal with samples of very different sizes.		

with using summary data from a database as "controls"			
1.2 You make use of a very good and reliable method to classify your variants as pathogenic or benign, or anything in between. Yet you do not yet integrate its results properly into your manuscript<br>2.3 The rationale for following up these 3 probands, and not the other 2 probands with NAGPA variants, remains somewhat weak	ED2 Apply the criteria for variant interpretation consistently in all cases and explain any departures from the standard analysis.		

2nd Review and Author's Response	21-Apr-2021

Reviewer(s)' Comments to Author:

Reviewer 1: Comments to the Author

I would like to thank the authors for working with my comments to adjust the manuscript. However, I have a few comments on the new additions, especially on the newly added comparison of allele frequencies.

Major comments

1. A new analysis was included in the manuscript to compare allele frequencies of the identified variants between the study cohort and the gnomAD database (Table 6).

- No details on the statistical analysis are provided in the material and methods section. Please add them.
- The huge difference in sample size between the study cohort and gnomAD make it difficult to properly compare the allele frequencies between the samples especially for rare variants. This is reflected in the significant results for several of the variants compared, for example GNPTG c.802A>C with frequencies 1/127 vs 1/30613 gives a highly significant result. Please use a statistical test that can deal with this difference in sample size, or correct by for example drawing random subsets of gnomAD with a similar sample size. You will see that 1/127 vs 0/127 does not give a significant difference.
- It is very difficult to do such a comparison with a public control dataset when looking at rare variants or variants with different allele frequencies between populations. Please describe properly what has been done to assure that the control cohort is comparable regarding ancestry before performing this analysis.

ANS: Table 6 and its related text is now removed from manuscript, as we understand that this kind of comparison with the controls from database is not appropriate.

2. You make use of a very good and reliable method to classify your variants as pathogenic or benign, or anything in between. Yet you do not yet integrate its results properly into your manuscript:

- In the results section on Family STU 63, you write "Since the de novo mutation occurred in a heterozygous condition in the affected, we assume that it is dominant." However, the variant is predicted to be benign. Please adapt accordingly.

ANS: The variant is not predicted as benign but a variant with uncertain significance. Since our study has confirmed it to be a *de novo* variant the classification is now 'likely pathogenic' but since we do not know the inheritance with respect to stuttering, we retain the pathogenic assertion as VUS.

- Results section, section about Family STU 34 (page 23, paragraph 3): You write that the pathogenicity of the variant p. Arg44Pro in NAGPA is inconclusive. However, according to table 2, this variant is "likely benign". Please adapt the text accordingly.

ANS: The variant is now rewritten as benign and reads as: (page 8)

“Mutation analysis identified a heterozygous missense variant p. Arg44Pro in exon 2 of *NAGPA* gene. Extended testing and analysis revealed the presence of this variant in his unaffected father and his two unaffected brothers (Figure 5). Also, ACMG classifies this variant as benign.”

- In the discussion section, page 26, paragraph 2, you discuss c.3598G>A in GNPTAB. You write that the high allele frequency of this variant in GnomAD “questions its pathogenicity.” In paragraph 3 on the same page, you write again about “conflicting evidence for pathogenicity”. However, in Table 2, this variant is classified as “benign”. Please adapt the text accordingly.

ANS: Yes, the text is uniformly adapted accordingly and reads now as: (Page 10)
“Recurrence of this lysine variant in heterozygous condition (0.8%) segregating with affected status in our study favors the founder effect in Asians. Though the lysine variant is highly conserved across species (Consurf =9) with pathogenic predictions and also *in silico* prediction of its effect on protein was shown to disrupt the helical segment which may be crucial for protein-protein interaction (figure A4), its high frequency among south Asian ancestry (2.1%) in the gnomAD database favours insignificance to stuttering pathology. Eight individuals in south Asian population also had this homozygous variant signifying that this variant is not lethal, as opposed to *GNPTAB* homozygous variants observed in mucolipidosis. Hence this lysine variant is confirmed to be benign in the present study”.

- In the discussion section, you discuss the c.131G>C variant in NAGPA. You mention “Since its frequency was also low in gnomAD database the role of this variant remains inconclusive.”. However, VarSome scored this variant as “likely benign”. Please adapt the text accordingly.

ANS: Yes, the text has been adapted accordingly and reads as: (Page 12)
Only one isolated case of European descent has been reported to have this variant among the stuttering population studied [21]. Observation of this variant in both affected and unaffected family members in our study suggests that this variant is less likely to have causal effect.

- The titles of Table 3 and Table 4 and a remark in Table 4 describe three variants as pathogenic variants. However, these variants are classified as benign or likely benign by VarSome. Please adapt accordingly.

ANS: It is adapted accordingly in the respective places. (Page 22).

Note: Due to shuffling and reframing of paragraphs and the order of tables and figures have changed.

Table 4: Variant profile of the probands in the three putative genes for stuttering

Table 5: Segregation pattern and genotype-phenotype correlation of variants identified in the putative genes for stuttering among three probands

Remarks: Cosegregation of the variant suggests a dominant inheritance pattern

Minor comments

1. At several locations (abstract paragraph 2; results section about Family STU 34) you write “p. Ile268Leu in GNPTG gene was found to be a de novo, thus reducing the allele frequency to 1.6% (2/128).” I don’t understand of what allele the frequency is lowered, and why.

ANS: This confusion is now addressed. Since there is only one pathogenic variant the likely causative allele frequency is 0.8% (1/128).

2. Page 19, paragraph 3: “The combined contribution of these genes were estimated to be 20% and all of them point to intracellular trafficking deficits(20).” Later articles have lowered this estimation to 12%. See for example Frigerio-Domingues C, Drayna D. Genetic contributions to stuttering: the current evidence. Mol Genet Genomic Med. 2017 Feb 19;5(2):95-102.

ANS: The reviewer suggest that later articles report 12% but I disagree because in that review article the author states 12% after discounting on some of the variants observed in population databases/control. But considering those individuals in the population databases as former stutterers, the estimate would remain as 20%. (Page 3)
The overall contribution of these genes was estimated to be 20% among the unrelated stuttering individuals. Though 8% of them was observed in public databases, considering the fact that they might not have been phenotyped for stuttering, they are likely to be current or former stutterers.

3. Page 19, paragraph 4: "However, family-based linkage studies till date did not report any significant signal at this location." Please explain in what locus. For example, the GNPTAB locus showed significant linkage with stuttering (Kang C, Riazuddin S, Mundorff J, Krasnewich D, Friedman P, Mullikin JC, Drayna D. Mutations in the lysosomal enzyme-targeting pathway and persistent stuttering. N Engl J Med. 2010 Feb 25;362(8):677-85.)

ANS: The phrase is reframed as below to clarify: (Page 3)
However, family-based linkage studies till date did not report any significant signal on chromosome 16p loci that bear *GNPTG* and *NAGPA* genes.

4. Page 20, paragraph 2: you describe a GWAS study on stuttering in 84 individuals. Please comment that p>10e-8 is not significant after correction for multiple testing.

ANS: The GWAS study mentions that the P value is significant and thus reported ten genes involved in neural pathways. Now we have introduced the clarification that the p value is significant in their study (Page 4).

"With limited statistical power, the study suggested ten candidate genes (*FADS2, PLXNA4, CTNNA3, ARNT2, EYA2, PCSK5, SLC24A3, FMN1, ADARB2* and a non-coding RNA *RNU6-259P*) with a significant p-value less than 10^{-4} and greater than 10^{-8}, involved in neural pathways".

5. The newly added text on segregation analysis and genotype-phenotype correlations is very long. Especially when describing three variants that are predicted to be benign or likely benign. Perhaps it can be summarized or partially moved to the supplement.

ANS: The newly added text on segregation analysis is now shortened, in the form of a summary.

6. You write "In fact, dominance and recessiveness are not essentially allelic properties but measured in relation to the effects of other alleles at the same locus." In the discussion section (page 26, last sentence). Please provide a reference for this statement, and perhaps more information, as it is a rather difficult concept.

ANS: The reference is already provided. Miko I. Genetic dominance: genotype-phenotype relationships. Nat Educ. 2008;1(1):140.

The following paragraph is added to bring clarity to the difficult concept. (Page 10)
The genotoype phenotype correlation is rarely simple as dominance and recessive patterns for a given gene as described by Mendel. In many cases multiple alleles contribute for a trait and includes variety of relationships between alleles (allelic interactions) that code for the same trait. Allelic dominance constantly depends on the relative influence of each allele responsible for the phenotype under given environmental conditions. In fact, dominance and recessiveness are not essentially allelic properties but measured in relation to the effects of other alleles at the same locus. Additionally, dominance may change according to the level of organization of the phenotype and its variations highlight the complexity of understanding genetic influences on phenotypes (ref 27).

7. You write in the conclusion that your mutation screening resulted in an allele frequency of 1.6%. Please detail of what allele this is the frequency. In case you mean "likely causal variants" or similar, please consider your VarSome results in Table 2.

ANS: Yes, now the likely causative allele frequency is corrected to 0.8% (1/128), since there is only one pathogenic variant.

8. In Table 1, please adjust the allele frequency of rs2937112 in the South Asian gnomAD: there is no data in this subset for this variant, so the allele frequency is not 0 but unknown.

ANS: Yes, in Table 1 the allele frequency is now stated as unknown and not '0'

Reviewer 2: Comments to the Author

The authors have made several updates to the paper, in response to the reviewers' comments. There are still a number of outstanding issues, detailed below. The most interesting finding in the paper remains the identification of the de novo variant in one proband, and the subsequent follow-up. The relevance of the other variants is unclear, and without suitable controls, summaries of the identified variants are very difficult to interpret.

- Please check again the gnomAD frequencies in table 1, and consider adding frequencies from 1000Genomes reference population too.

ANS: Yes we agree. With variants where gnomAD frequency is unknown, 1000 genome frequency is now added in the table 1.

For example, Variant 4 (c.139C>T in NAGPA) should have an allele count of 92. This site is multiallelic, and the count for a different allele is given :

https://gnomad.broadinstitute.org/variant/16-5083677-G-A?dataset=gnomad_r2_1

ANS: Yes we agree and the necessary correction are now made.

o Variant 12 (c.1174+53C>A in NAGPA): The gnomAD frequency in South Asians, does indeed appear to be 0; so to see so many probands with this variant seems strange. Looking at dbSNP, in the 1000Genomes population, this SNP has an allele frequency of 0.57 (A) in the South Asian population. (I am unsure why the frequency in gnomAD was 0.) https://www.ncbi.nlm.nih.gov/snp/rs2937112#frequency_tab

ANS: We have checked by revisiting the databases and it is given in this manner and so we also feel the same discrepancy with respect to the two databases. Perhaps the filters used by the two databases may be varying.

- I have concerns about the addition of table 6 and what this adds. Firstly, there is no description of the statistical methods use – how were the p-values calculated? Secondly, there are several issues with using summary data from a database as "controls" – see https://doi.org/10.1101/115964 for discussion. This table was seemingly added in response to the comment by both reviewers, that the authors do not provide evidence that more variants would be seen in the probands, than would be expected by chance. I do not think table 6 sufficiently provides this evidence, and it would be better if the authors remove this from the manuscript.

ANS: Yes we agree. Table 6 and its related text is now removed from manuscript, as we understand that this kind of comparison with the controls from database is not appropriate.

- The additional detail about the three probands is interesting; The rationale for following up these three probands, and not the other 2 probands with NAGPA variants, remains somewhat weak. It is (vaguely) stated that follow-up was based on conservation score and frequency.

ANS: The rationale for follow up of the three probands was based on high conservation score and pathogenic prediction in both SIFT and Polyphen. To be honest the segregation analysis was limited to prioritized variants due to restraints in funds. This whole project was run without any proper funds but squeezed from different projects acknowledged in manuscript.

The results state "Also the gnomAD exome allele frequency is greater than the required threshold confirming their benign nature.", but "the required threshold" is not stated anywhere.

ANS: This statement is now removed to avoid confusion.

In the discussion it states "Three of the conserved nonsynonymous variants with less than 1% MAF for segregation analysis that are discussed individually" – so perhaps the threshold is 1%? However, c.3598G>A in GNTAB has a MAF>2% in individuals of South Asian ancestry in gnomAD. Please clarify further criteria for follow-up (I assume this particular variant was selected for follow-up, given that specific variant was previously reported?).

ANS: Yes, c.3598G>A variant in GNPTAB was selected as it was previously reported. We agree that MAF criteria is not applicable for all the three variants and hence now removed.

Minor:

• Introduction (page 19 in proof):

o "The Genetic epidemiological studies provided inconsistent evidence on the modes of inheritance, yet revealed familial clustering of the disorder and may be monogenic in a subset of cases and families, rendering stuttering as a complex genetic trait" – this sentence seems a little contradictory of itself?

ANS: Clarity is now given and reads as: (Page 3)
Though familial aggregation was observed genetic epidemiological studies provided inconsistent evidence for mode of inheritance, with varied reports on dominant, recessive and sex-modified inheritance. Genetic dissection is challenging due to gene-gene/gene-environment interactions, genetic heterogeneity, gender bias, incomplete penetrance and phenocopies.

And
o "However, family-based linkage studies till date did not report any significant signal at this location." – I'm unclear what this is referring to?

ANS: The phrase is reframed as below to clarify: (Page 3)
However, family-based linkage studies till date did not report any significant signal on chromosome 16p loci that bear *GNPTG* and *NAGPA* genes.

• Results (page 21):

o "The identified variants and their interactions" – this sounds like the authors are suggesting interactions (biological or statistical) – this should be re-worded.

ANS: Yes it is re-worded: the word interactions is replaced by the word combinations: (Page 5) and it now reads as follows:
The identified variants and their combinations were analyzed in an upset plot using R program.

• Legend for table 3 is "Variant profile for the three putative genes for stuttering in probands with pathogenic mutation" – remove mention of "pathogenic mutation".

ANS: The title of the table 3 is corrected as below: (Page 22)
Table 4: Variant profile of probands in the three putative genes for stuttering

Editorial Decision on second revision	21-Apr-2021

Your manuscript which you revised in light of two rounds of peer review and editorial comments and resubmitted to Advanced Genetics, is now accepted in principle pending minor revisions that I have requested. Please see marked up manuscript and checklists for journal requirements for acceptance.

Author response	02-May-2021

Thank you for accepting our manuscript in principle after two rounds of revision. We have uploaded the final revised manuscript with minor revisions that includes reframing of title as given above. We have also added the URL links and related papers in the references that enhances the list from 39 to 54.

Editor Decision: Accept	03-May-2021
